# Stimuli-responsive vesicles as distributed artificial organelles for bacterial activation

**DOI:** 10.1073/pnas.2206563119

**Published:** 2022-10-12

**Authors:** Ignacio Gispert, James W. Hindley, Colin P. Pilkington, Hansa Shree, Laura M. C. Barter, Oscar Ces, Yuval Elani

**Affiliations:** ^a^Department of Chemical Engineering, Imperial College London, South Kensington, London, SW7 2AZ, UK;; ^b^fabriCELL, Imperial College London, Molecular Sciences Research Hub, White City, London W12 0BZ, UK;; ^c^Department of Chemistry, Imperial College London, Molecular Sciences Research Hub, White City, London W12 0BZ, UK;; ^d^Institute of Chemical Biology, Imperial College London, Molecular Sciences Research Hub, White City, London W12 0BZ, UK

**Keywords:** artificial cells, artificial organelles, membranes, synthetic biology, cell signaling

## Abstract

Intercellular communication is a hallmark of living systems. As such, engineering artificial cells that possess this behavior has been at the heart of activities in bottom-up synthetic biology. Communication between artificial and living cells has potential to confer novel capabilities to living organisms that could be exploited in biomedicine and biotechnology. However, most current approaches rely on the exchange of chemical signals that cannot be externally controlled. Here, we report two types of remote-controlled vesicle-based artificial organelles that translate physical inputs into chemical messages that lead to bacterial activation. Upon light or temperature stimulation, artificial cell membranes are activated, releasing signaling molecules that induce protein expression in *Escherichia coli*. This distributed approach differs from established methods for engineering stimuli-responsive bacteria. Here, artificial cells (as opposed to bacterial cells themselves) are the design unit. Having stimuli-responsive elements compartmentalized in artificial cells has potential applications in therapeutics, tissue engineering, and bioremediation. It will underpin the design of hybrid living/nonliving systems where temporal control over population interactions can be exerted.

## Significance

Artificial cells are entities designed to mimic biological cells in form and function. One of the grand challenges of this field is engineering “nonliving” artificial cells to communicate with their biological “living” counterparts. Combining the programmability of tailor-made artificial cells with the biotechnological power of biological cells has the potential to underpin applications in biotechnology and medicine. By achieving on-demand externally controlled activation of biological cells, the artificial cells constitute programmable modules that translate physical inputs into chemical signals that bacteria respond to. Crucially, the bacteria gain an extended sensory range without undergoing genetic engineering. This paves the way toward assembling artificial organelles and opens up new avenues in using artificial cells as tools in therapeutic applications and beyond.

Artificial cells are engineered mimics of biological cells, constructed from the bottom up by bringing together defined molecular building blocks (1). They are designed to replicate the form, function, and behaviors of natural cells, and most often are based on enclosed compartments that contain biomolecular species responsible for imparting cell-like features (compartmentalization, sense/response, communication, etc.) (2, 3). In addition to being used as simplified cell models to decipher the rules of life through an “understanding by building” approach (4), a major motivation behind artificial cell research is their potential to act as devices that can be used in biomedical and biotechnological applications (5–7).

There are several advantages associated with artificial cells compared with genetically engineered biological cells that are the preserve of top-down synthetic biology. This includes the ability to incorporate nonbiological functional components, reduced regulatory and biosafety considerations, and the removal of cell-burden limitations that arise when engineered cellular functions exist alongside native ones (8). However, perhaps the major attraction of artificial cells is that they only contain the minimal components required to perform their function and can thus be composed of only a small number of molecular species. It is therefore easier to engineer them to have user-defined features, so they are highly controllable and programmable. However, their reduced complexity means that they cannot currently match the metabolic, regulatory, and behavioral sophistication of their biological counterparts. Processes in biological cells may thus be more robust, with cells able to perform their functions even in the presence of perturbations or changes in their milieu (9), which is often the basis of their use in many applications (10).

The programmability of artificial cells and the technological potential of biological cells have led to increased efforts at coupling the two together, and in the process, accumulating the advantages associated with both (11). This can be achieved by either forming hybrid living/artificial cells (12–14) or engineering communication routes between populations of living cells (bacteria or eukaryotes) and artificial ones (15–20). In so doing, not only could the extensive genetic modification of cells be bypassed (as the artificial cells are the engineered species), but also more complex functions could be reconstituted when both types of cells are incorporated into the same system.

Integrating artificial and biological cells requires establishing communication pathways between them, for example, through quorum sensing using autoinducer molecules (21, 22). This approach was exploited to expand the sensory range of bacteria through the construction of artificial cells that operated as chemical translators that could sense a small molecule and release an inducer molecule that bacteria could respond to (23). Similarly, robust artificial cells were designed to act as sensor or reporter modules depending on the DNA program present in the artificial or bacterial cell (24). In recent years, engineered communication has been extended to artificial-eukaryotic cell networks (17, 25–27).

Nevertheless, the current systems rely on chemical triggers, while the use of physical stimuli remains mostly unexplored. We intended to remedy this by engineering stimuli-responsive artificial cells which can communicate with bacteria.

In so doing, stimuli-responsive artificial cells would constitute distributed “artificial organelles,” which are not encapsulated within living cells but endow bacteria with novel responsiveness to physical stimuli. This would also provide artificial/living hybrid cell systems with a control step to allow external activation by an end user on demand. Designing a library of stimuli-responsive platforms entails the next breakthrough in cellular bionics, since controlling cell behaviors using artificial cells as intermediaries would enable processes to take place in a temporally controlled manner, in a defined location, and in response to specific stimuli. There have been several examples of stimuli-responsive population communication operating between synthetic cell populations, including communication between cell-like compartments in synthetic tissues using a light-regulated DNA promoter (28), through divalent cation chelators (29), or using a photolabile DNA linker (30). In a recent exciting preprint, a light-activated DNA template was used to control communication between synthetic and bacterial cells using ultraviolet (UV) light (31). Nevertheless, to realize the incorporation of hybrid artificial/living cell systems in applications such as therapeutics, bioremediation, and biosensing, remote-controlled cell mimics able to communicate with a wide range of biological cells, using different stimuli, need to be further developed (32). Herein, we address this gap by designing a generalizable vesicle-based artificial cell platform capable of compartmentalizing content, sensing a physical cue, and releasing chemical messengers that can then activate a DNA program in *Escherichia coli*.

To do this, we developed and exploited two types of synthetic cell compartments, one of which was light responsive and the other thermoresponsive. These house inducer molecules with them, which are ordinarily shielded from surrounding bacteria. Only upon encountering the relevant stimuli do these molecules get released and activate protein expression in the living cells. This allowed us to control protein expression in cells using artificial cells and intermediaries that translate the stimuli (light and heat) into a chemical signal that the cells respond to.

## Results

### Stimuli-Controlled Artificial-Bacterial Cell Activation.

**Fig. 1.**
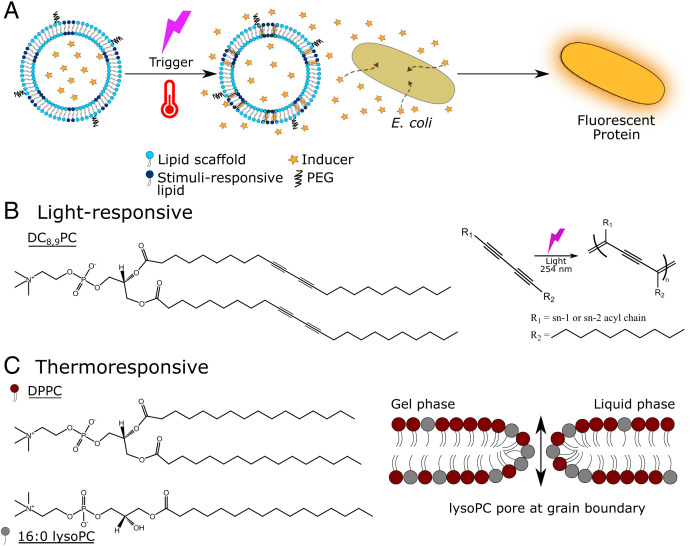
Controllable activation of bacteria using light- or temperature-responsive artificial organelles. (*A*) Cartoon of stimuli-triggered activation. Vesicle-based artificial organelles are assembled containing a stimuli-responsive lipid within the lipid membrane. Upon external stimulation, pores open in the membrane, and the encapsulated cargo is released, thus inducing protein expression in *E. coli.* (*B*) Structure of DC_8,9_PC and photopolymerization reaction upon light (254 nm) irradiation. The photopolymerization of DC_8,9_PC causes the formation of pores at the grain boundaries of the lipid membrane. (*C*) Structure of DPPC and 16:0 lysoPC. The presence of 16:0 lysoPC in the lipid membrane promotes the formation of pores at the grain boundary between gel and liquid phase lattices when the temperature approaches the T_m_.

We have created two different nanoscale vesicle-based artificial cells, which when exposed to light irradiation or temperature, release their encapsulated content. The light-responsive vesicles were constructed via thin-film hydration followed by extrusion, with the photopolymerizable lipid DC_8,9_PC (which has been employed in drug delivery (33) and in nested vesicle enzymatic reactors (34)) embedded in a matrix of 1,2-distearoyl-sn-glycero-3-phosphocholine (DSPC), a common component of lipid formulations employed in drug delivery. 1,2-distearoyl-*sn*-glycero-3-phosphoethanolamine-*N*-[methoxy(polyethylene glycol)-2000] (DSPE-PEG 2000) was also incorporated to increase the stability of the final formulation (35). To create robust artificial cells that are stable in bacterial cultures and showcase optimal release properties, the final formulation was 87:10:3 DSPC:DC_8,9_PC:DSPE-PEG 2000. The pivotal component of our formulation is DC_8,9_PC, which does not mix with the rest of the components but instead forms segregated lipid domains that result in patches (36) in the membrane. Upon light irradiation (λ = 254 nm) DC_8,9_PC photopolymerizes (Fig. 1*B*), entailing the production of defects at the domain boundaries that cause the formation of pores (36–38) through which the encapsulated content is released.

The thermoresponsive artificial cells are composed of a 1,2-dipalmitoyl-sn-glycero-3-phosphocholine (DPPC) matrix and 1-palmitoyl-2-hydroxy-sn-glycero-3-phosphocholine (lysoPC). During the last two decades, these lipids together with DSPE-PEG 2000 have been used to construct thermosensitive liposomes for combined drug delivery and hyperthermic treatment for cancer (39–41). DPPC possesses a melting temperature (T_m_) around 41.5 °C, which enables mild hyperthermic release. The addition of low amounts of lysoPC enhances the permeability of lipid bilayer membranes at the gel-to-liquid phase transition (42), resulting in a higher and faster release (43). It has been proposed that lysoPC promotes the opening of toroidal pores at the grain boundaries (44–46), between liquid and gel domains when the temperature approaches the T_m_ (Fig. 1*C*). The ability to quickly release the encapsulated content—without compromising the integrity during activation experiments—is fundamental when designing thermoresponsive artificial cells. Accordingly, the final composition was 90:10:4 DPPC:lysoPC:DSPE-PEG 2000, which has been shown to work in vitro (47) and in vivo (48), exhibiting quick release and good stability for drug delivery applications.

**Fig. 2.**
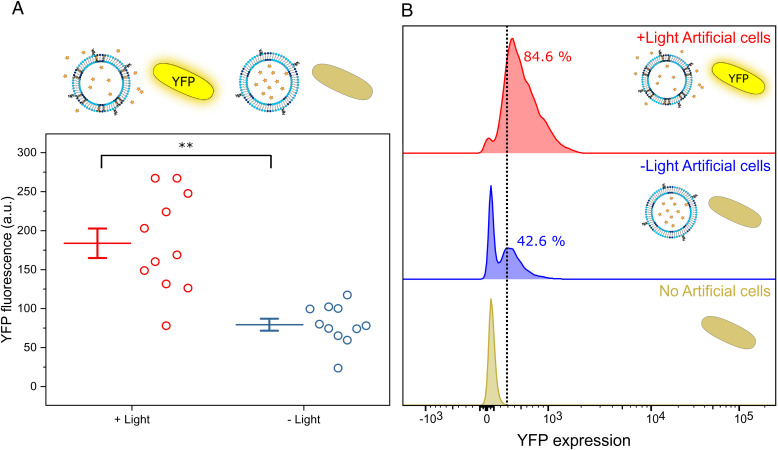
Activation of bacterial cells using light-responsive artificial organelles. (*A*) YFP production upon irradiation. Fluorometry results depict how irradiated artificial cells (red, *Left*) achieve a twofold increase in YFP production compared with expression using nonirradiated artificial cells (blue, *Right*). Each data point corresponds to independent experiments. Solid lines represent the mean, error bars correspond to 1 SEM (*n* = 11). *P* values calculated using the 99.5% confidence interval (**P < 0.005). (*B*) Histograms of YFP expression obtained using flow cytometry. *E. coli* cells without added artificial cells (brown, *Bottom*) are used as a control for their intrinsic fluorescence. The addition of irradiated artificial cells increases the number of bacterial cells expressing YFP from ∼43% with nonirradiated artificial cells (blue, *Middle*) to ∼85% (red, *Top*).

In our experiment, two different stimuli-responsive vesicle-based artificial organelles have been developed to mediate the activation of bacterial cells. For the light-responsive system, the signaling molecule that is encapsulated in the vesicle is isopropyl β- d-1-thiogalactopyranoside (IPTG), which induces the activation of the lac operon circuit in *E. coli* (49). This enabled light-induced activation of yellow fluorescent protein(YFP)-expressing *E. coli*; the YFP expression is only triggered when IPTG is released from the artificial cells (Fig. 1). The artificial cells were prepared by rehydrating a lipid film with 100 mM IPTG, 20 mM Hepes, pH 7.4 buffer extruded using 100 nm filters and purified by size exclusion chromatography (SEC). Then, 40 min of irradiation was employed to release the encapsulated IPTG before being added to *E. coli* cultures (Optical Density (OD) ∼1) to induce YFP expression. After 24 h incubation at 20 °C, the activation was assayed using a fluorometer (Fig. 2*A*) and flow cytometry (Fig. 2*B*) measuring YFP production.

As depicted in Fig. 2*A*, a twofold statistically significant (*P* < 0.005; *P* value calculated using the confidence interval of the + light/– light ratio, *n* = 11 independent protein expression experiments) fluorescence increase was achieved when using irradiated artificial cells (+ light, red) instead of nonirradiated (– light, blue), thus, demonstrating the possibility of establishing an effective light-triggered activation of living cells using artificial cells. The observed variability between the fluorometric measurements within the same group was equivalent, according to an F-test of equality of variances, to that observed in samples induced with standard concentrations of IPTG (*SI Appendix*, Fig. S1).

To obtain population-level insights into our system, the same samples measured with fluorometry were analyzed using flow cytometry. The histograms depicted in Fig. 2*B* are a representative example of light-triggered YFP expression. Using cells incubated without IPTG-loaded artificial cells (green) as a control for background fluorescence signal, it was found that when using nonirradiated vesicles (– light artificial cells, blue), ∼43% of the *E. coli* would express YFP while the incorporation of irradiated vesicles (+ light artificial cells, red) duplicates (∼85%) the number of cells responding. The histograms also enable the analysis of the total fluorescence of the sample, using the area under the curves. Analysis of the histogram curves (*SI Appendix*, Fig. S2) presents a twofold statistically significant increase in fluorescence, (*P* < 0.005; obtained using the confidence interval, *n* = 10) when using irradiated artificial cells (+ light, red) instead of nonirradiated (– light, blue). These results obtained from analyzing single cells are in accordance with the fluorometry measurements that correspond to the whole population. Thus, both measurement techniques confirm that enhanced YFP expression is achieved using light-activated artificial cells, indicating that a user-controlled light stimulus is able to trigger the release of IPTG from the artificial cells on demand.

Our vesicle-based artificial cells are stable in the growth medium employed for the incubation (see dynamic light scattering [DLS] measurements in *SI Appendix*, Fig. S3). A calcein (428 mOsm/kg) release assay (*SI Appendix*, Fig. S4) shows passive release in lysogeny broth (LB) growth medium (osmolarity = 220 mOsm/kg), whereas no release is detected in 500 mM sucrose buffer (510 mOsm/kg) or when LB is supplemented with 500 mM sucrose (LB + sucrose, 530 mOsm/kg). No release is detected in the presence of bacteria or their supernatant (*SI Appendix*, Fig. S4), and the release properties remain identical after 5 d of storage (*SI Appendix*, Fig. S5)

Light provides high spatiotemporal control of the process and can be applied topically; however, it suffers from poor tissue penetration (even if using infrared light) (50) and attenuation in aqueous environments. On the other hand, heat is easier to apply and has greater translational power. Thus, using thermoresponsive artificial organelles to control temperature-activated protein expression could enable a wider variety of applications involving activation in deep tissue.

**Fig. 3.**
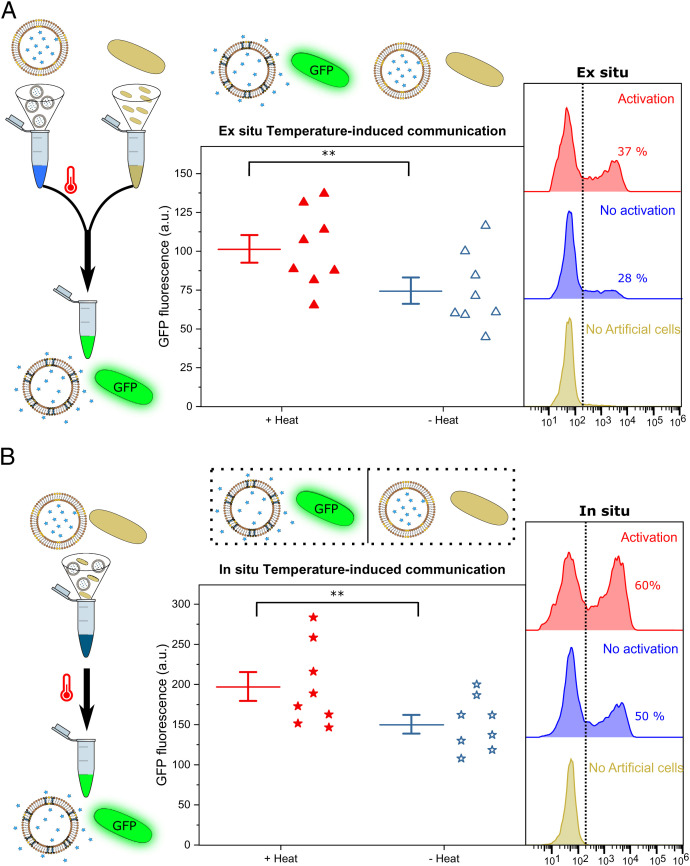
Temperature-controlled activation of protein expression in bacteria using artificial organelles. (*A*) Ex situ activation. The artificial cells are heated to release the inducer before being added to the bacteria. Fluorometry results depict how heated artificial cells (red triangles, *Left*) achieve a 1.4-fold increase in GFP production compared with expression using nonirradiated artificial cells (blue open triangles, *Right*). The histograms obtained using flow cytometry show that the addition of heated artificial cells increases the number of bacterial cells expressing GFP from ∼28% with nonheated artificial cells (blue, *Middle*) to ∼37% (red, *Top*). *E. coli* cells alone (brown, *Bottom*) are used as a control for their intrinsic fluorescence. (*B*) In situ activation. Both artificial cells and bacteria are mixed together before the activation step. Fluorometry analysis of GFP expression of systems where the artificial cells have been activated (red stars, *Left*), or not (open blue stars, *Right*), shows a 1.3-fold increase in GFP production upon activation. Flow cytometry analysis shows that activated samples of artificial cells (red, *Top*) induce expression of GFP in 60% of the bacterial cells. Nonactivated samples (blue, *Middle*) show 50% of cells expressing GFP. Each data point corresponds to independent experiments. Box plots: solid lines represent the mean, error bars correspond to 1 SEM (*n* = 8). *P* values calculated using the 99.5% confidence interval (**P < 0.005).

The thermoresponsive artificial organelles were employed to activate *E. coli* with a superfolder green fluorescent protein (sfGFP)-expressing plasmid encoded under the control of a rhamnose promoter. Rhamnose-loaded vesicles release the inducer for green fluorescent protein (GFP) expression in *E. coli* exclusively upon heating (Fig. 3). Before that, however, standard concentrations of rhamnose were employed to induce GFP expression in bacterial cells subjected to 43 °C for 5 min (as for in situ activation) or kept at 37 °C (as for ex situ activation) (*SI Appendix*, Fig. S6). The levels of GFP expression were similar for both configurations, and no statistical difference was found in heating ex or in situ (*P* > 0.05 for all rhamnose concentrations, calculated using an unpaired *t* test, *n* = 6), thus, confirming that the trigger for the activation does not affect the bacteria. The artificial cells were prepared following a similar protocol as above: lipid film rehydration with 1,000 mM rhamnose, 20 mM Hepes, pH 7.4 buffer, followed by extrusion using 100 nm filters and SEC purification. Both ex situ and in situ experiments were conducted to determine the feasibility of both configurations, seeking to understand the possible differences that may arise from heating the system. For ex situ experiments, the artificial cells were heated for 5 min at 43 °C to release the encapsulated rhamnose, before being added to *E. coli* cultures (OD ∼0.1) to induce GFP expression. Whereas during experiments in situ, the artificial and living cells were mixed together before the activation step.

Using once again the protein expression as a measure to determine the activation, after 4 h incubation at 37 °C, the samples were analyzed using a fluorometer and flow cytometry. When conducting experiments ex situ (Fig. 3*A*), heated thermoresponsive artificial cells induced a GFP fluorescence signal 1.4-fold higher than when the artificial cells were not heated (*P* < 0.005; *P* value calculated using the confidence interval, *n* = 8). During in situ experiments (Fig. 3*B*), heated samples had a GFP expression 1.3 times higher than when there was no heating (*P* < 0.005; obtained with the confidence interval, *n* = 8). The variability observed in the GFP fluorescence intensities obtained upon ex situ and in situ activation was equivalent, according to an F-test of equality of variances, to that observed in samples induced with standard concentrations of rhamnose (*SI Appendix*, Fig. S7).

The analysis using flow cytometry, with samples of bacterial cells alone as a control (brown histogram), revealed that using ex situ conditions (Fig. 3*A*), 37% of the bacterial cells were expressing GFP after the trigger was applied (red), while 28% expressed GFP when they were not activated (blue). Further analysis of the area under the curve of the histogram (*SI Appendix*, Fig. S8) corroborated the observed increase (1.5-fold, *P* < 0.005; calculated using the confidence interval, *n* = 8) in the total fluorescence of the samples. When the in situ (Fig. 3*B*) samples were analyzed, 60% of the bacterial cells expressed GFP upon heating (red histogram) and 50% of them did when not heated (blue). These translated into a 1.3-fold difference (*P* < 0.005; calculated using the confidence interval, *n* = 5) of the area under curve (*SI Appendix*, Fig. S8).

All the data confirm the successful activation of bacteria using thermoresponsive vesicle-based artificial organelles to control the process. Both ex situ and in situ activations are feasible and effective; hence, thermoresponsive units display a versatile behavior that could be implemented to mediate bacterial responses in a wide range of settings.

However, as it was observed with the light-responsive network, populations of bacterial cells where the artificial cells have not been activated produce an increase in fluorescence. Therefore, the leakiness of the artificial cells in the growth medium was determined using a calcein release assay (*SI Appendix*, Fig. S9). The growth medium for GFP expression is M9 buffer (see composition details in *Methods*), and its osmolality is 130 mOsm/kg, whereas the encapsulated calcein employed during the release assay is 425 mOsm/kg, as determined by freezing-point depression using an osmometer. This difference is strong enough to cause the passive release from the inside of the artificial cells. To counterbalance the internal osmotic pressure, M9 was supplemented with more fructose yielding a buffer, isosmotic to the internal calcein, that lowers the passive release from the artificial cells (*SI Appendix*, Fig. S9).

We repeated the artificial cell-mediated protein expression experiments using M9 buffer isosmotic with the internal rhamnose solution. This buffer, M9+, has an osmolality of 1,126 mOsm/kg compared with the internal rhamnose’ osmolality of 1,120 mOsm/kg. The artificial cells were stable in M9+, as indicated in the DLS measurements in *SI Appendix*, Fig. 10. The temperature-induced GFP expression in M9+ was performed with in situ heating due to its more favorable outlook for further applications. Analysis of the GFP expression using a fluorometer (*SI Appendix*, Fig. S11) revealed that the heated artificial cells produce a twofold increase (*P* < 0.05; calculated using the confidence interval, *n* = 6) in the GFP signal compared with nonheated samples. Expression in M9+ buffer increases the difference between the heated and nonheated samples (twofold in M9+ vs. 1.3 for M9); however, the GFP fluorescence is 10 or 14 times lower for the heated or nonheated conditions, respectively, when using M9+ instead of M9 (*SI Appendix*, Fig. S12). This can be explained because the growth of the bacterial cells in M9+ is slower, and the maximum OD is 25% lower than when using M9 (*SI Appendix*, Fig. S13). The expression using standard concentrations of rhamnose was also lower (*SI Appendix*, Fig. S14), especially for high rhamnose concentrations (>10 mM), thus indicating that protein expression is hindered by the higher osmotic stress. Despite the improvement observed with M9+, the obtained fluorescence intensities were very low and impeded further analysis. However, these experiments confirm that the osmolarity should be considered when designing future artificial-living cell networks (51).

### Characterization of Light-Responsive and Thermoresponsive Artificial Cells.

The bacterial activation, measured as protein production, depends on the available inducer for the bacteria. Characterization of release rates ex vivo is hence critical to understanding how to externally modulate the controlled activation of artificial cell–bacterial networks. To investigate the release efficiency from the artificial cells, 50 mM calcein was encapsulated as a model small signaling molecule within the vesicles in 500 mM sucrose, 20 mM Hepes, pH 7.4 buffer before triggering content release using the appropriate stimulus.

**Fig. 4.**
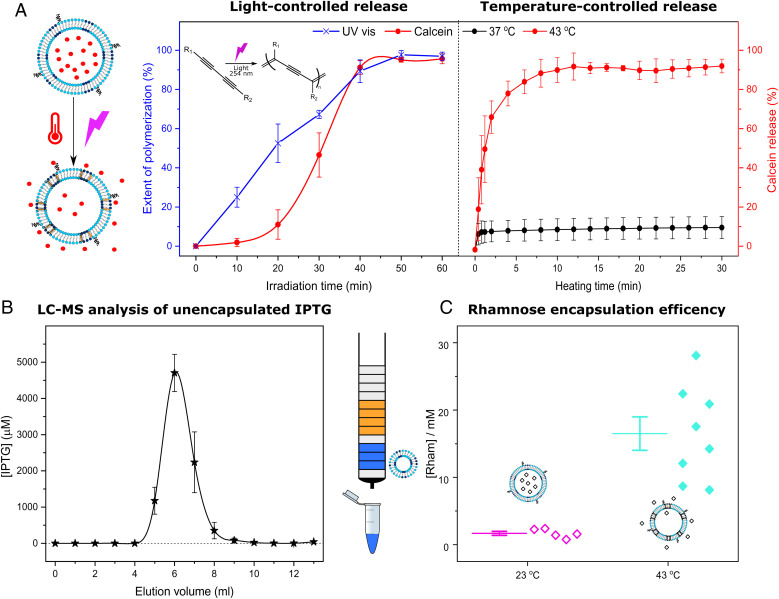
Encapsulation efficiency and triggered content release from the artificial cells. (*A*) 254 nm light produces the photopolymerization of DC_8,9_PC, which can be followed (blue) by measuring the absorbance peak appearing at 475 nm. Photopolymerized DC_8,9_PC forms pores, resulting in the release of calcein (*Left panel*, red, normalized data using Eq. 1 from *Methods*). The thermoresponsive lipid vesicles release their cargo through the pores that are generated at the grain boundaries when gel and liquid phases coexist near the T_m_ (43 °C, *Right panel*, red). At lower temperatures (37 °C, *Right panel*, black), the lysoPC does not form pores. Error bars show 1 SD, (*n* = 3). (*B*) Encapsulation efficiency for the light-responsive composition is estimated by measuring unencapsulated IPTG with LC-MS. When purifying the artificial cells using SEC, free unencapsulated IPTG appears in the fractions that elute after the vesicles. The IPTG in the fractions eluting after the artificial cells is measured at [M + H]^+^ = 239. Error bars show 1 SEM, (*n* = 3). (*C*) The encapsulation and the purification efficiency of the temperature-sensitive artificial cells are determined using an enzymatic kit to measure rhamnose. Vesicles purified via SEC were analyzed before (23 °C, pink) and after (43 °C, cyan) stimulation.

Fig. 4*A* (*Left panel*) presents the light-mediated calcein release (red circles). During the initial irradiation, before 20 min, little calcein is released; however, during the next 20 min, a sharp increment from 10 to 90% occurs. The addition of Triton detergent enables determining the 100% release and shows that the maximum release obtainable by irradiation is 90%. This incomplete release could be attributed to the presence of ∼5% multilamellar vesicles (*SI Appendix*, Fig. S15, *Left*), as well as to small variations in the vesicle-to-vesicle composition that result in different release properties.

When DC_8,9_PC is removed from the formulation (DSPC:DSPE-PEG 2000 97:3), no release is observed after 60 min irradiation (*SI Appendix*, Fig. S16), therefore proving that DC_8,9_PC is required for light-mediated release to occur. As previously reported elsewhere (36–38), release occurs through pores opening at the boundaries between the segregated DC_8,9_PC domains and DSPC. To confirm this, light-responsive vesicles were measured using DLS before and after 60 min of irradiation (*SI Appendix*, Fig. S3). The vesicles did not lyse upon light irradiation, as the shape and mean diameters remained unchanged. The stimulus effect was further explored employing cryo-transmission electron microscopy (cryo-TEM), which showed that DSPC:DC_8,9_PC:DSPE-PEG 2000 (87:10:3) vesicles present a faceted morphology (*SI Appendix*, Fig. S15, *Left*) before and after light irradiation, as previously reported (52). We hypothesize that the faceted appearance occurs due to the gel phase nature of this lipid composition (53) and the presence of segregated DC_8,9_PC domains. Thus, the cryo-TEM experiments further reinforced that the light-mediated release must occur through pores opening in the membrane.

Low (10 mol%) DC_8,9_PC concentrations can hence be used to rapidly modulate membrane permeability in the presence of light irradiation. We note that this time would be rapidly reduced with increasing light intensity (experiments here used a standard handheld 4-watt [4W] lamp).

When DC_8,9_PC photopolymerizes, the formation of ene-yne conjugates results in a broad peak appearing in the 440–540 nm region of the absorbance spectrum (54) (*SI Appendix*, Fig. S17). Therefore, UV-visible spectroscopy can be used to follow the extent of photopolymerization. As presented in Fig. 4*A* (crosses in blue), the photopolymerization occurs faster than the release of calcein. We hypothesize that a minimum degree of polymerization may be necessary to create the discontinuities that lead to the formation of pores at the domain boundary edges between DC_8,9_PC and DSPC.

On the other hand, the thermoresponsive composition provides a much quicker response to the stimulus with >85% of calcein releasing within 5 min when heated at 43 °C (Fig. 4*A*, *Right panel*, red circles). At 37 °C, this thermoresponsive composition is stable, showing less than 8% release (Fig. 4*A*, *Right panel*, black circles), even after 12 h of incubation (*SI Appendix*, Fig. S9, red line), which endows our compositions with better retention than other commercially available compositions (55). Other temperatures below 43 °C produce lower release (*SI Appendix*, Fig. S18), and their total induced release never matches that resulting from heating to 43 °C. Nevertheless, the release properties at 43 °C do not change after 5 d of storage (*SI Appendix*, Fig S19). Further optimization of the manufacturing process could enable full release by further increasing the unilamellarity of the thermoresponsive artificial cells from the current ∼95% (*SI Appendix*, Fig. S15, *Right*).

In the absence of 16:0 lysoPC (DPPC:DSPE-PEG 2000 96.2:3.8), no release is detected until the heating temperature reaches 41 °C, when ∼6% of calcein release is observed (*SI Appendix*, Fig. S20). The drastic difference in the release has been linked to the role of lysoPC in the stabilization of grain boundaries in the lipid bilayer. Raising the temperature of the lipid bilayer increases its fluidity. As temperatures approach the T_m_, adjacent lattice grains may have different fluidity levels, some remaining in a solid phase while others have already melted. In this situation, nanopores are formed at the boundaries, and the encapsulated content releases through them. Cryo-TEM confirmed that the membranes were not disrupted during the heating process (*SI Appendix*, Fig. S15, *Right*).

Both compositions, with and without lysoPC, were further characterized using differential scanning calorimetry (DSC). The thermograms (*SI Appendix*, Fig. S21) show the main temperature-dependent phase transition at ∼41–43 °C. In accordance with previous reports (44), the presence of lysoPC causes the disappearance of the pretransition peak of DPPC at 36.5 °C, mainly due to the disruption that lysoPC induces in the ordered bilayer membrane phase of DPPC.

In addition to using calcein to test release rates from the different vesicles, the encapsulation efficiency of the inducers was estimated to determine the loading concentrations of the chemical signals in large unilamellar vesicles. The light-responsive network employs IPTG, and it is a poor chromophore and fluorophore; thus, we used liquid chromatography–mass spectrometry (LC-MS) to estimate the encapsulation efficiency in the artificial cells. A direct measurement of the encapsulated IPTG was not possible because the size of the vesicles could block the pores in the LC-MS column. Consequently, we prepared 50 mM IPTG artificial cells, and we purified them using SEC, where the artificial cells elute before the unencapsulated IPTG fractions. These unencapsulated IPTG fractions were measured as [M + H]^+^ = 239 after adapting a protocol published by Fernández et al. (56) (see characteristic chromatograms of standard concentration samples in *SI Appendix*, Fig. S22). Fig. 4*B* shows the detected signal for each fraction of the SEC purification column. To avoid blocking the chromatography column, neither the vesicle fractions (fractions 2–4) nor the two fractions that followed (5, 6) were analyzed. The rest of the eluted fractions from the SEC column containing unencapsulated IPTG were analyzed using LC-MS. The total IPTG concentration remaining unencapsulated was calculated using a calibration curve (*SI Appendix*, Fig. S23) and corresponded to 35% of the initial used to prepare the artificial cells; thus, the encapsulation efficiency of our system is ∼65%.

The thermoresponsive network employs rhamnose to induce the expression of GFP in *E. coli*. Rhamnose can be measured using a commercial enzymatic kit consisting of L-rhamnose dehydrogenase and NAD^+^. Rhamnose is oxidized by NAD^+^ in the presence of L-rhamnose dehydrogenase to L-rhamno-1,4-lactone, yielding NADH, which is measured at 340 nm by UV-vis spectroscopy. This increase is stoichiometric with the amount of L-rhamnose. A calibration curve (*SI Appendix*, Fig. S24) was prepared and employed to determine the encapsulation in thermoresponsive artificial cells. We employed 1,000 mM rhamnose to hydrate the lipid films as described in *Methods*. After SEC purification, thermoresponsive artificial cells were heated to 43 °C for 5 min and subsequently cooled down to 23 °C in a water bath. Then, aliquots with the appropriate volume were assayed using the rhamnose enzymatic kit (Fig. 4*C*, cyan rhombus). The encapsulation efficiency was determined to be ∼1.6% in accordance with previous reports (48). Furthermore, this rhamnose assay can be employed to determine the efficacy of SEC purification by measuring the free rhamnose present in eluted vesicles that have been kept at 23 °C, when no release has occurred. The data in Fig. 4*C* (pink rhombus) indicate that only 1.5 mM of the initial 1,000 mM rhamnose was detected. This would indicate that the vesicles have been purified with a 99.85% efficacy.

Finally, the release at different temperatures using the light-responsive system was also determined to investigate whether the activation could occur at physiological temperatures (*SI Appendix*, Fig. S25). At 37 °C, 50% of passive release was detected, while at 20 °C the release was negligible. To better understand this thermal effect, DSC scans (*SI Appendix*, Fig. S26) were run to identify the thermally induced phase transitions of DSPC:DC_8,9_PC:DSPE-PEG 2000 (87:10:3). The thermogram presented the main transition at ∼53 °C and also two pretransitions at 37 °C and 39 °C, which were also observed by Yavlovich et al. (36) and could be associated with the transition of DC_8,9_PC (occurs at 43 °C), hence explaining the cargo release at 37 °C. Therefore, the light-responsive system requires lower temperatures to prevent the activation via temperature. As shown above, at the ideal conditions for protein expression, light can be used for ex situ control, while the thermoresponsive artificial cells endow in situ control.

## Discussion

Communication between artificial and biological cells has been applied in varied fields, including biomedicine for the production of antimicrobial peptides (24) and antitumoral (57) proteins, bioremediation to extend the range of pollutants that can be tackled (58, 59), and fuel cells to treat waste water (60, 61). However, to further leverage the use of bacteria in biotechnology, vesicle-based artificial organelles can be incorporated as a tool to extend the responsiveness and functionalities of biological cells.

Here, we have designed a generalizable platform that enables the use of a physical stimulus (light or temperature) to control the activation of bacteria. Both light and temperature offer the possibility to induce protein expression ex situ, where both modules are physically separated, and, in the case of temperature, in situ, where both artificial and living cells coexist in the same space. The ex situ activation, where the signal is produced in a different space than the response, emulates endocrine signaling, while the in situ activation replicates paracrine signaling, with artificial and living cells coexisting in the same physical space before and during the process.

We have characterized our system using a combination of spectroscopy-based methods, including a calcein release assay to demonstrate the functionality of the lipid formulation, DLS, and an enzymatic assay and mass spectrometry to determine the encapsulation efficiency. After ensuring the stability (in the growth medium) and adequacy (encapsulation efficiency of the inducers) of the artificial cells to induce protein expression, fluorescence spectroscopy and flow cytometry were employed to assess the expression obtained using activated vesicles. Using the light-induced artificial cells, a twofold increase in the produced YFP was recorded when using irradiated vesicles compared with nonirradiated vesicles, whereas temperature-induced activation generates a 1.4-fold increase in GFP expression when employed ex situ and a 1.3-fold increase if applied in situ.

Light-mediated activation experiments were undertaken at 20 °C, bypassing the cargo release resulting from the gel to fluid transition in the DSPC:DC_8,9_PC:DSPE-PEG 2000 membrane and also enabling expression on the bench, which could be desired in the future depending upon the protein of interest (62, 63). For example, it is common to obtain inclusion bodies (insoluble protein aggregates) when overexpressing proteins at 37 °C using *E. coli* (64).

Our stimuli-responsive vesicle-based artificial organelles could be integrated into multicompartmentalized (65–67) hybrid systems between artificial and biological cells, where each unit performs different functions. Considering that compartmentalization is essential to perform bio-orthogonal processes (67), multistep catalytic reactions (68, 69), and in vitro transcription and translation (70), conferring increased levels of spatiotemporal control, could enable complex reactions and processes in a step forward toward creating artificial organelles, especially if the in situ control that the thermoresponsive artificial cells provide, even at physiological temperatures, is exploited. For this purpose, we envision the coencapsulation of artificial and living cells inside giant vesicles to create new hybrids. Moreover, the use of physical stimuli such as light and temperature opens up the possibility of exerting spatial and temporal control and may allow the degree of activation (and extent of response) to be modulated by tuning signal input parameters (signal intensity and duration).

Finally, this work constitutes a generic framework for on-demand activation of artificial cell/biological cell communication, where artificial cells are programmed to act as translator modules converting physical stimuli to chemical ones which living cells can respond to. This is a wholly generalizable platform that can potentially be extended to any stimuli-responsive membrane system in the future. This distributed approach (22, 23), where stimuli-responsive elements are compartmentalized in artificial cells and not embedded in living ones, represents an alternative to the genetic modification of living systems, offering increased control over cell composition without the concerns of unwanted environmental persistence common for genetically engineered systems. Cellular protein expression is controlled here through the addition of just a single synthetic chemical functional group or a lysolipid to the artificial cell, highlighting the potential for the bottom-up construction of artificial cells approach to biodesign.

## Methods

### Production of Lipid Vesicles.

All lipids were purchased from Avanti Polar Lipids, Inc. Vesicles were prepared by thin-film rehydration. First, a lipid mixture was prepared from CHCl_3_ lipid stocks stored at –20 °C. The mixture was vigorously mixed for 2 min to ensure homogeneity, and then the remaining CHCl_3_ (l) was evaporated under a stream of N_2_ (g) to create dry lipid films, which were kept overnight under vacuum.

Vesicle dispersions were created by rehydrating the lipid film with an appropriate buffer. Then, light-responsive vesicles were subjected to four freeze-thaw cycles, while eight cycles were used for thermoresponsive vesicles.

SUVs were prepared by passing the lipid dispersion 21 times through a 100 nm polycarbonate filter using an Avanti Mini Extruder heated above 60 °C. The extruded vesicles were kept for 30 min at 4 °C to reverse possible activation produced when heated. Finally, purification was achieved using an SEC column of Sephadex G-50 as follows. First 0.4 g of Sephadex G-50 were hydrated with 12 mL of the appropriate elution buffer for 1 h at 60 °C. Then, the plastic column was assembled and loaded with the hydrated Sephadex. After an equilibration period of 30 min, the extruded vesicle solution was poured dropwise. The elution buffer was added in fractions of 300 µL, and the purified sample was collected.

### Release from Vesicles.

Light-responsive vesicles were placed in an open 96-well plate at ∼7.5 cm and were irradiated using a UVG-11 compact UV lamp (254 nm shortwave UV emission, 230 V, 4W) at room temperature. Thermoresponsive vesicles were heated directly in the fluorimeter or using a Thermomixer C with 1.5 mL microcentrifuge tubes.

### Quantify Release Efficiency Using Calcein.

Calcein vesicles were prepared by rehydrating the lipid film using 50 mM calcein, 20 mM Hepes, 100 mM KCl, pH 7.4 buffer. For the purification column, a 500 mM sucrose, 20 mM Hepes, 100 mM KCl, pH 7.4 buffer was employed. To quantify the release upon different irradiation times, calcein fluorescence was recorded at λ_ex/em_ = 494/514 nm using a Cary Eclipse Fluorometer (Agilent Technologies). The total release was calculated by lysing nonactivated vesicles using the detergent Triton X-100. Then, the release efficiency (%) was calculated using Eq. 1, where F_S_ is the fluorescence at a specific irradiation time, F_0_ is the fluorescence of nonirradiated vesicles before lysis, and F_T_ is the fluorescence after adding Triton X-100.

[1] $$\mathrm{Release}\;\mathrm{efficency}\;\left(\text{\%}\right)=\frac{F_{s}-F_{0}}{F_{t}-F_{0}}\;\cdot 100$$

IPTG was analyzed using a Waters Acquity UPLC (Milford) liquid chromatograph coupled to electrospray ionization triple quadrupole Waters Xevo TQ-MS mass spectrometry. Using electrospray ionization in positive mode, the acquisition conditions were as follows: capillary at 3.2 kV, cone at 25 V and 25 l·h^−1^, source temperature at 150 °C, and desolvation temperature at 400 °C with 25 l·h^−1^ N_2_ (g). The column, Rezex RHM-Monosaccharide H+ (8%), 300 × 7.8 mm (Phenomenex), was kept at room temperature during the analysis. The mobile phase used was LC-MS Grade Water with 0.1% formic acid running in isocratic mode at a flow rate of 0.6 mL·min^−1^. A 1 h equilibration step was required before any measurement was performed. We injected 10 µL of the sample for a total run time of 20 min. For the first 11 min, samples were diverted from the mass spectrometer; then, they were injected into it for 4 min (11–15 min). Finally, for the last 5 min, samples were rediverted. IPTG was detected at 12.6 min by monitoring *m/z* 239 [M + H]^+^ and *m/z* 261 [M + Na]^+^. Vesicles were prepared with 50 mM IPTG as described above. After the vesicles appeared in the SEC column, 8 × 1000 µL fractions that contained the unencapsulated IPTG were collected. These 8 × 1,000 µL samples were subsequently analyzed with LC-MS.

Encapsulation of rhamnose was determined by employing a commercial enzymatic kit from Megazyme. In the presence of L-rhamnose dehydrogenase, NAD^+^ oxidized rhamnose to L-rhamno-1,4-lactone, producing NADH. The standard microplate protocol was employed as follows: 210 µL of distilled water was added to a 96-well, clear, flat-bottomed plate; then, 10 µL of buffer 1 (pH 10), 10 µL of NAD^+^ (with polyvinylpyrrolidone, bottle 2), and 10 µL of the sample (either standard rhamnose solution or vesicles) were added. After 3 min of incubation at 25 °C, 5 µL of the enzyme wase added, and the formation of NADH was monitored over 20 min at 340 nm using a VarioSkan Flash microplate reader (Thermo Fisher). Standard concentrations of rhamnose (0–20 mM) were employed to construct a calibration curve that was used to determine the unencapsulated rhamnose after SEC and its release from thermoresponsive artificial cells.

### *E. coli* Transformation.

BL21 *E. coli* (Code C2527l, New England BioLabs) was used to express YFP, while GFP expression was performed using *E. coli* BW25113 (CGSC# 7636). The genetic construct pAJM.336 (Addgene plasmid #108528), employed for YFP expression under lac operon control, was a gift from Christopher Voigt (Massachusetts Institute of Technology) . Rhamnose-controlled GFP expression was achieved using plasmid pAB410, kindly gifted by Alice Boo (Imperial College Lonodn).

Competent *E. coli* were transformed to express YFP using the heat-shock method: one vial (0.05 mL) of competent cells was thawed on ice, and 1.5 µL of plasmid was added and mixed by gently tapping the vial. After 30 min incubating on ice, to heat-shock the cells, the vial was introduced in a water bath at 42 °C for 10 s and then returned to the ice for another 5 min. Then 950 µL of Super Optimal broth with Catabolite repression (SOC) medium at room temperature was added to the vial, and it was incubated for 1 h at 37 °C, 250 rpm.

Finally, serial dilutions of the cell suspensions were plated in LB plates containing kanamycin (200 µg·mL^−1^) and were incubated overnight at 37 °C. The next day, single colonies were picked to make 5 mL cultures with LB supplemented with kanamycin. After overnight incubation at 37 °C, 250 rpm, 50% (vol/vol) glycerol stocks were prepared for long-term storage at –80 °C. Glycerol stocks of *E. coli* BW21113 already transformed with construct pAB410 were received and stored at –80 °C.

### Protein Expression in *E. coli.*

For YFP expression, an overnight culture was prepared from a glycerol stock and was incubated overnight at 37 °C, at 220 rpm, in an orbital shaker under aerobic conditions using LB supplemented with kanamycin (200 mg/l). The next day, the culture was diluted 50 times in fresh LB with kanamycin and grown until OD∼0.5. Next, bacteria were harvested by centrifugation (4,400 rpm, 10 min) using a Rotanta 430R centrifuge (Hettich) and resuspended to OD∼1 in LB with kanamycin and 100 mM sucrose. Expression occurred at 20 °C, 220 rpm in 50 mL falcon tubes with 5 mL of cells supplemented with 0–100 mM IPTG (Panreac AppliChem). For vesicle-induced expression, vesicles were prepared as described above using 100 mM IPTG, 20 mM Hepes, 100 mM KCl, pH 7.4. After irradiation, vesicles were added to the falcon tubes maintaining a total culture volume of 1 mL

GFP was expressed in BW25113 cells, which were grown overnight at 37 °C, 220 rpm, in an orbital shaker under aerobic conditions from glycerol stocks. M9 media consisting of M9 minimal salts (5×) supplemented with 0.4% casamino acids, 0.25 mg/mL thiamine hydrochloride, 2 mM MgSO_4_, 0.1 mM CaCl_2_, 0.4% fructose, and 60 µg/L spectinomycin was employed. In the morning, cultures were diluted to OD∼0.05 in the appropriate M9 media and grown until OD∼0.1. We transferred 100 µL of cells to clear 96-well plates; then, the appropriate amounts of rhamnose were added to each well, and more M9 was used to ensure a final volume of 200 µL. Breath-easy membranes (Sigma) were employed to mitigate evaporation during expression at 37 °C, 220 rpm. For vesicle-induced expression, vesicles were prepared as described above using 1,000 mM rhamnose and 20 mM Hepes. For in situ activation experiments, equal volumes of vesicles and cells were mixed in 1.5 mL centrifuge tubes and heated for 5 min at 43 °C before being placed in the 96-well plate. The final volume was kept constant at 200 µL.

### Protein Expression Analysis.

Two methods were used to analyze the protein expression: fluorometry and flow cytometry. For YFP fluorometry, 200 µL of cells were placed in 96-well plates, and expression was monitored at λ_ex/em_ = 514/524 nm using a Cary Eclipse Fluorometer. GFP expression was monitored at λ_ex/em_ = 485/528 nm using a Cary Eclipse Fluorometer or a CLARIOstar Plus plate reader (BMG Labtech,).

YFP analysis with flow cytometry was conducted using a BD LSRFortessa Cell Analyzer (BD Biosciences) using a blue laser (488 nm) with a 525/50 filter, whereas an Attune Nxt Flow Cytometer (Thermo Fisher) equipped with a 96-well plate autosampler was chosen for GFP analysis (488 nm blue laser, 530/30 filter). Data analysis was completed using FlowJo software from BD Biosciences.

Different statistical tests have been employed in this article. When establishing stimuli-controlled activation of bacteria, the confidence interval for the +activation/–activation ratio was determined (α = 5, 0.5, or 0.05%). The *P* value was then equal to the level of significance (α) whose confidence interval satisfied that the mean +activation/–activation ratio minus the confidence interval was >1.

To compare the variability in the protein expression, F-tests of equality of variances were employed to compare the expression with standard rhamnose concentrations with the expression mediated by vesicles. The mean values for expression using rhamnose standards were compared using an unpaired *t* test.

### Characterization of Lipid Artificial Cells.

DLS was employed to measure the vesicle size. We further diluted 50 μL of vesicles in 450 μL buffer, and their size distribution was measured using a Zetasizer nanoparticle characterization system (Malvern Panalytical). The lipid phase transitions of the membrane were studied using DSC. Stainless steel pans (TA Instruments) were filled with anhydrous lipid samples (8–12 mg) and 60–70 wt% water before being sealed. Two temperature cycles were taken from 20 to 70 °C or 25–55 °C, at a rate of 1 °C/min (heating and cooling) on a PerkinElmer Diamond DSC. The osmotic pressure was measured using a freezing-point depression osmometer (Micro-Digital, Löser i-Osmometer Basic M).

The polymerization of DC_8,9_PC was measured at 475 nm using a NanoDrop 2000 spectrophotometer. Lipid vesicles were prepared using 20 mM Hepes, 100 mM KCl, pH 7.4 buffer, and their absorbance spectra, 190–840 nm, were recorded at different irradiation times. Three drops were measured for each sample with three repetitions for each drop.

### Cryogenic Transmission Electron Microscopy.

Cryo-TEM was used to determine the size and morphology of stimuli-responsive lipid-based artificial cells. Vesicles were generated and heated/irradiated using the same methodology described above. Samples (3 µL; ∼5 mg/mL) were transferred onto a carbon grid (Quantifoil R 2/2 on 300 copper mesh; Jena Bioscience), in a Vitrobot (ThermoFisher) at 90% humidity. Sample grids were blotted (blot time: 7 s; blot force: 0; wait time: 32 s) and then immediately plunged into liquid ethane bath contained within a liquid nitrogen reservoir. The grid was transferred to a grid holder at –180 °C. Grids were transferred under liquid nitrogen to an electron microscope sample holder (Cryo Transfer Tomography Holder, Eden Instruments; Model 2550). Defocus values (generally between –0.5 and –5 µM, depending on the chosen magnification) were determined elsewhere on the grid to avoid sample damage from the electron beam. The instrument and camera used were FEI Tecnai12 BioTwin 120kV with TVIPS XF416 4K CMOS detector. Image handling and analysis were carried out using ImageJ software, subjecting each image to a Gaussian blur filter.

## Supplementary Material

Supplementary File

## Data Availability

All study data are included in the article and/or supporting information.
