# Supplementary material for: Stimuli-responsive vesicles as distributed artificial organelles for bacterial activation
Source: Proc Natl Acad Sci U S A. 2022 Oct 12;119(42):e2206563119. doi: 10.1073/pnas.2206563119 (PMC9586261; doi:10.1073/pnas.2206563119)
Supplement: Supplementary File [file pnas.2206563119.sapp.pdf]

PNAS

Supporting Information for

Stimuli-responsive vesicles as distributed artificial organelles for bacterial activation

Ignacio Gispert[a,b,c,d], James W. Hindley[b,c,d]*, Colin Pilkington [a,b,c], Hansa Shree[c], Laura Barter[c,d], Oscar Ces[b,c,d]* and Yuval Elani[a,b]*

Corresponding authors: James W. Hindley, Oscar Ces, Yuval Elani

Email: j.hindley14@imperial.ac.uk, o.ces@imperial.ac.uk, y.elani@imperial.ac.uk

This PDF file includes:

Figures S1 to S26

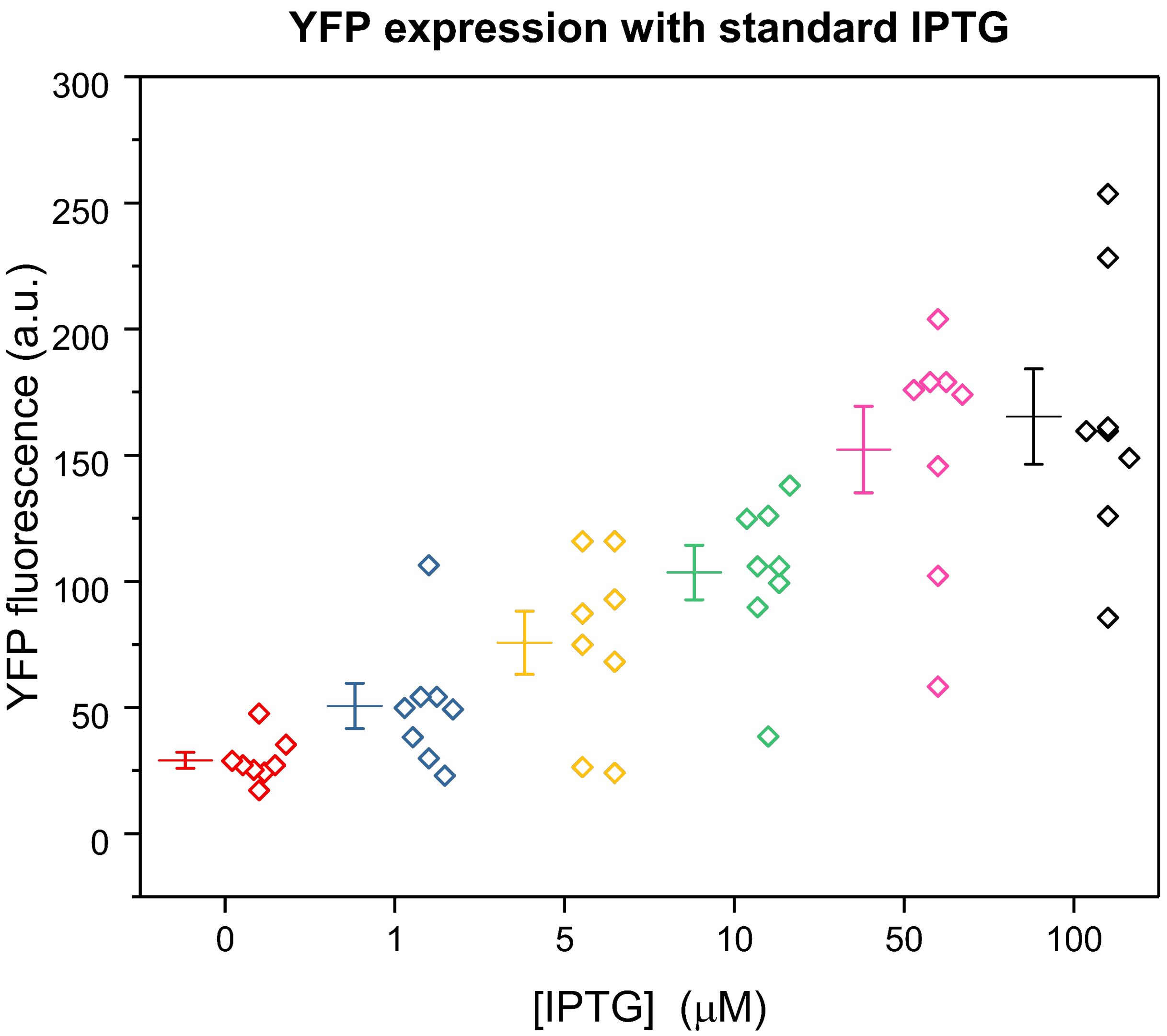

Figure S1. YFP expression using standard concentrations of IPTG. Increasing concentrations of IPTG were employed to induce YFP expression in E. coli. An F-test of equality of variances was conducted to determine if the variability in the results observed when using irradiated artificial cells was the same as when using standard concentrations of IPTG. For all the concentrations that achieved significant expression (≥5 μM), the variability was equivalent (F-test, P values of 0.101, 0.052, 0.326, 0.425 when using 5, 10, 50, 100 μM IPTG). Solid lines represent the mean, error bars correspond to 1 S.E.M (n=8).

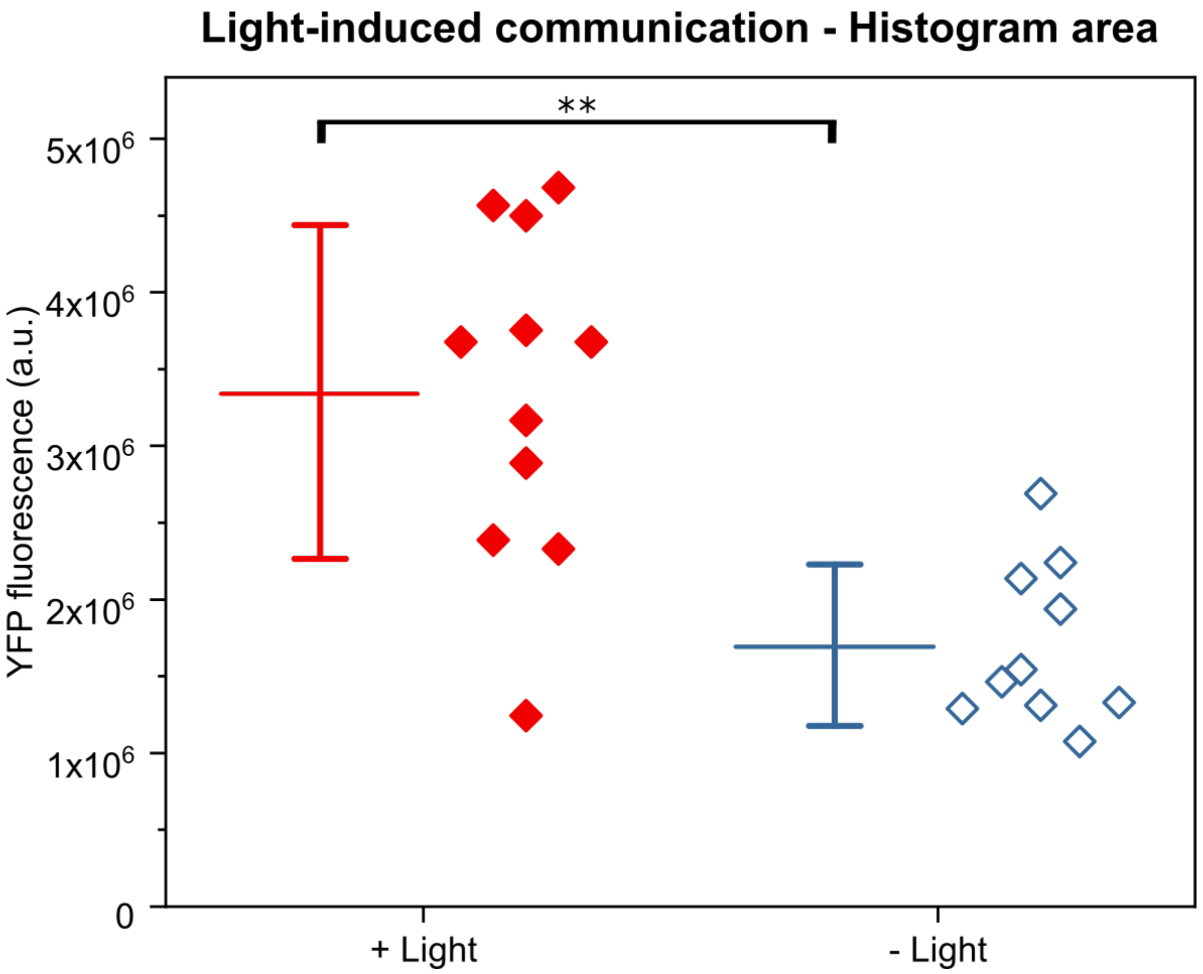

Figure S2. YFP expression as the area under the histogram curve obtained using flow cytometry. The total fluorescence of a sample can be calculated as the sum of each cell's fluorescence i.e. the area under the YFP expression histogram curve. Samples induced with irradiated artificial cells (red, left) show a 2-fold increase in YFP fluorescence compared to samples induced using non-irradiated artificial cells (blue, right). Each data point corresponds to independent experiments. Solid lines represent the mean, error bars correspond to 1 S.E.M (n=10). P-value calculated using the 99.5 % confidence interval (**P < 0.005).

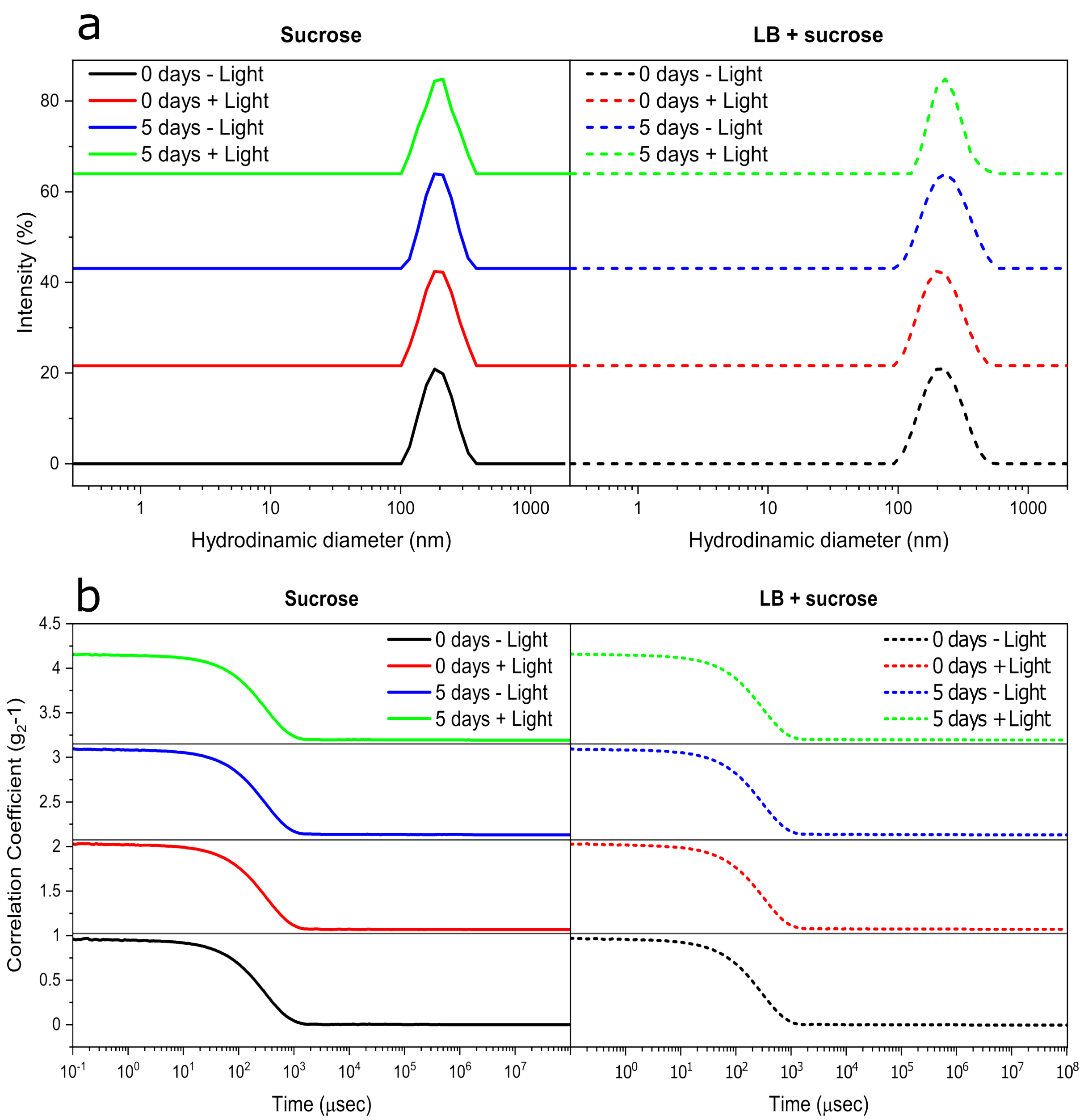

Figure S3. DLS measurements of artificial cells in the bacterial growth medium for light-induced activation. **a)** Stability over 5 days is observed in samples diluted with 500 mM sucrose buffer (left) and in the growth medium (LB + sucrose, right). Mean diameters increased from 202.6 nm to 208.1 nm when in sucrose buffer and from 204.7 nm to 222.5 nm in the growth medium. All the samples show a uniform size distribution as indicated by the polydispersity indexes with values starting at 0.03 and 0.08 when in sucrose or the growth medium just after preparation (day 0). After 5 days the values were 0.02 and 0.12. No changes were observed when the vesicles were irradiated for 60 min (red and green) in sucrose buffer or the growth medium. **b)** Correlograms of the DLS measurements show a single exponential decay which corresponds to mono-size particle dispersions.

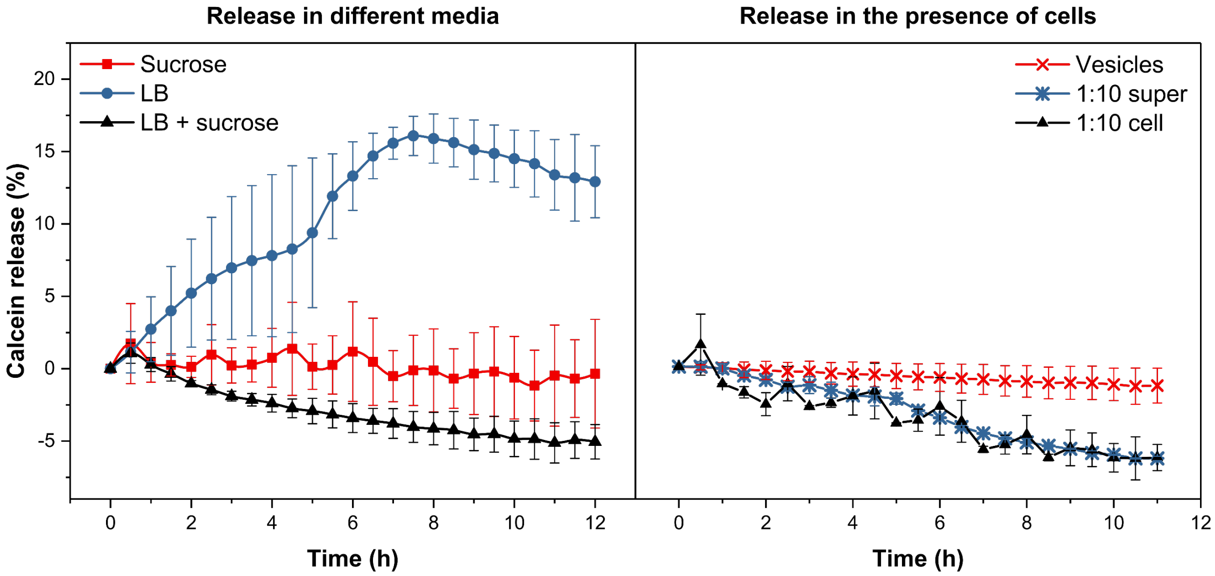

Figure S4. Light-responsive artificial cells do not release their cargo in the growth medium nor with bacteria or their supernatant. 50 mM calcein was encapsulated inside the artificial cells and the release overnight was determined. **a)** When the artificial cells are incubated with LB (blue dots), ~17 % of calcein releases overnight. Because LB is hypoosmotic to the encapsulated calcein, LB = 220 mOsm/kg and calcein = 428 mOsm/kg. By supplementing the LB with sucrose, i.e. the growth medium, (black triangles) calcein leakage is prevented (osmolarity = 530 mOsm/kg) and also occurs in a 500 mM sucrose buffer (red). **b)** Release from samples incubated with cells in LB + sucrose (black) or in the supernatant of a cell culture grown using LB + sucrose (blue). In both cases, the calcein release is negligible. Error bars show 1 SD. (n = 3).

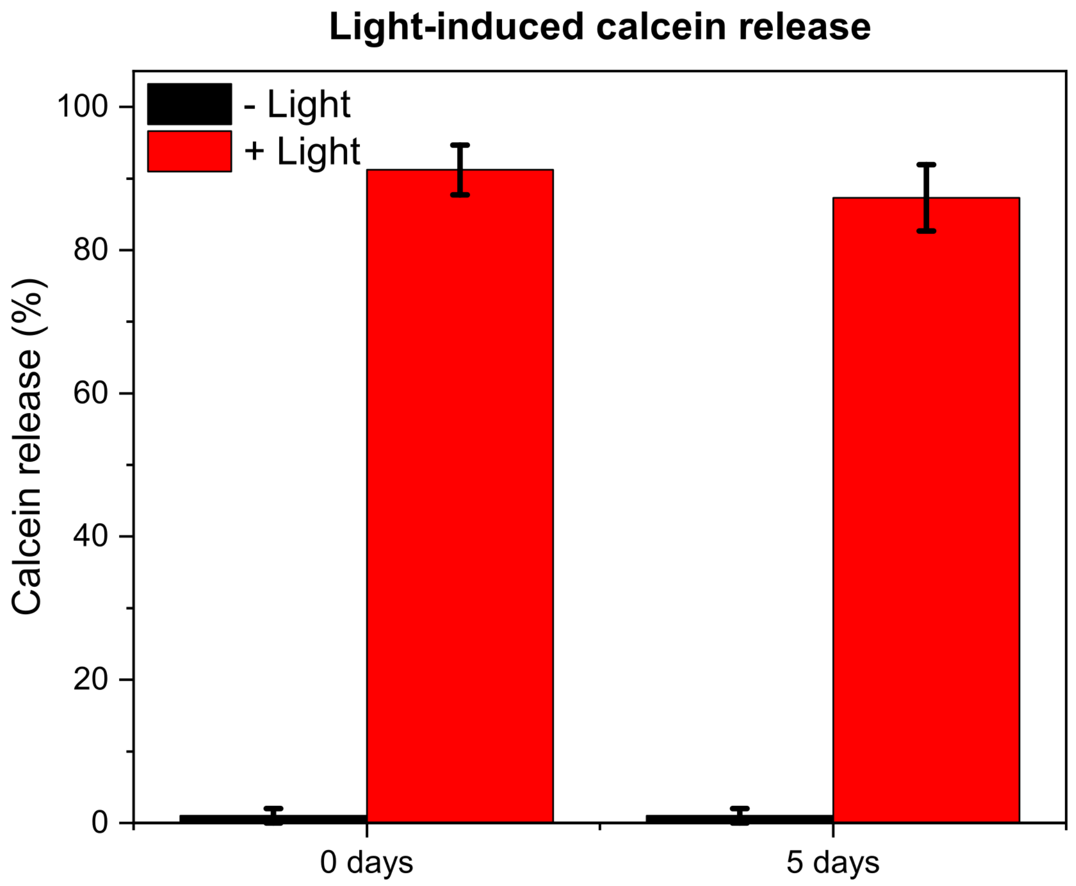

Figure S5. Light-responsive artificial cells show the same release after 5 days of storage. Calcein release was measured after 40 min of irradiation (red) and, after storage at 4 °C for 5 days, the release assay was repeated. No difference was observed in the release efficiency. The release without light irradiation was 0% in both cases. Error bars show 1 SD, (n = 3).

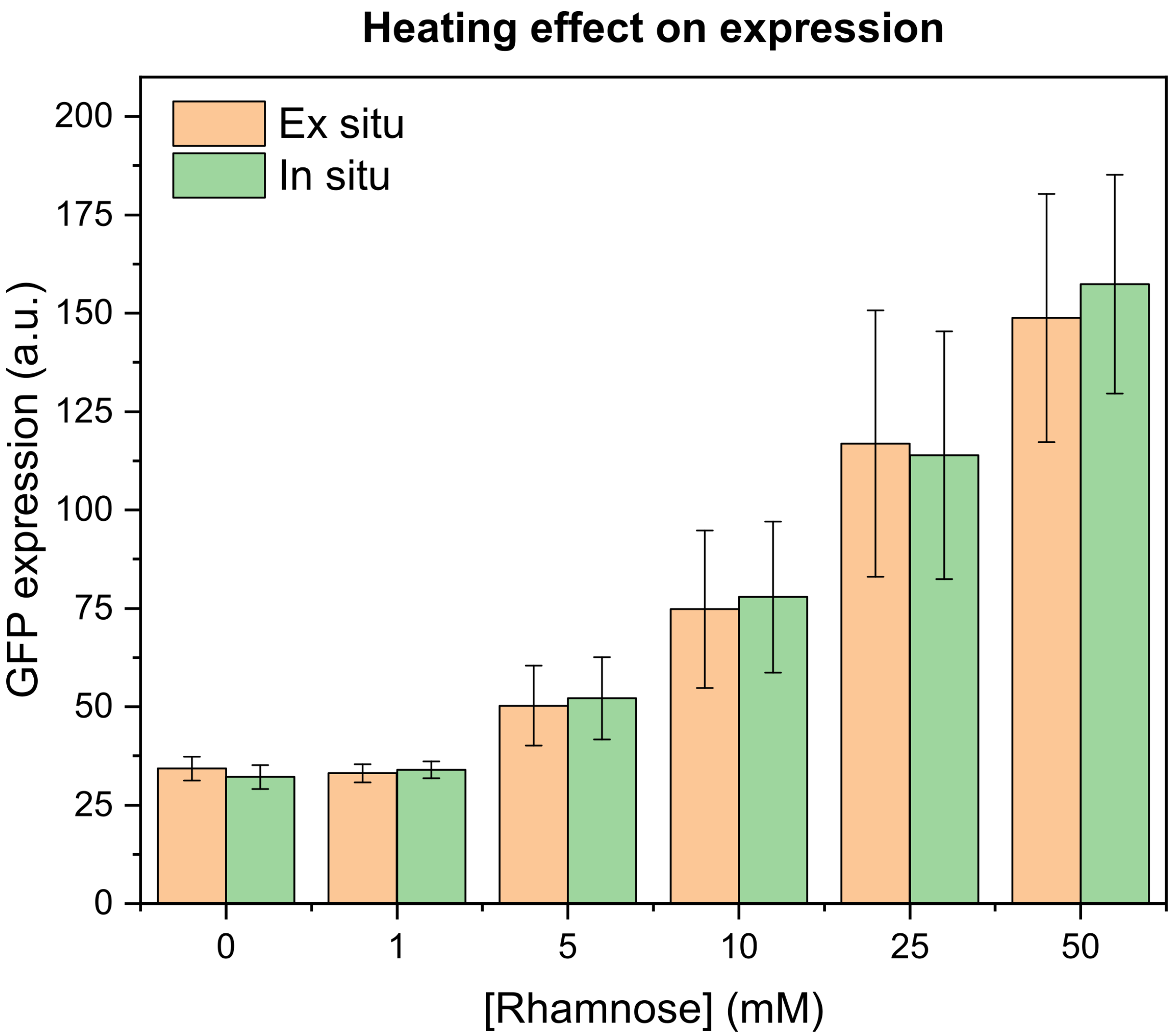

Figure S6. The activation trigger could affect the protein expression in bacterial cells when applied *in situ*. After 4 h of incubation at 37 °C, the GFP expression using standard concentrations of rhamnose is equivalent when the cells have been previously heated at 43 °C for 5 min (as for *in situ* activation, green columns) or when the bacterial cells have not been heated (as for *ex situ* activation, orange columns). p values calculated using an unpaired t-test: p=0.304, 0.574, 0.756, 0.787, 0.891, 0.893 for 0, 1, 5, 10, 25, 50 mM rhamnose. Error bars show 1 SD. (n = 6).

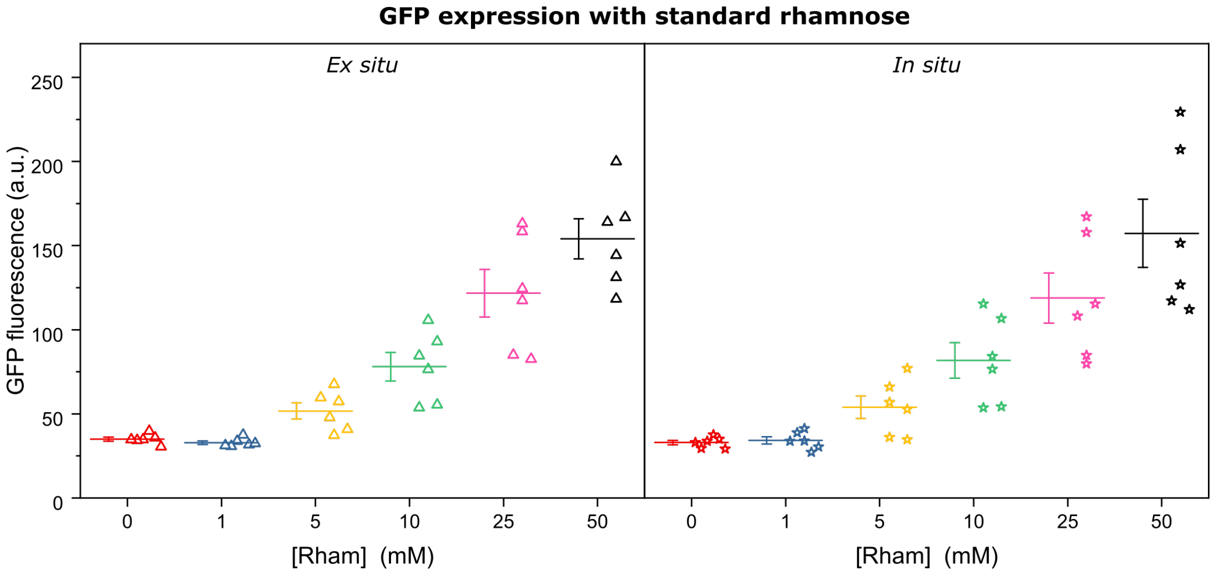

Figure S7. GFP expression using standard concentrations of rhamnose. Increasing concentrations of rhamnose were employed to induce GFP expression in *E. coli*, measurements were taken after 4 h of incubation at 37 °C. The variability in the GFP fluorescence from bacterial samples induced with standard concentrations of rhamnose or with activated artificial cells is equivalent according to an F-test of equality of variances. For all the concentrations that achieved significant expression (>5 mM), the variability was equivalent when heating *ex situ* (P values of 0.297, 0.294, 0.436 when using 10, 50, 100 mM rhamnose) and *in situ* (P values of 0.056, 0.190, 0.421 when using 10, 50, 100 mM rhamnose). Each data point corresponds to independent experiments. Solid lines represent the mean, error bars correspond to 1 S.E.M (n=6).

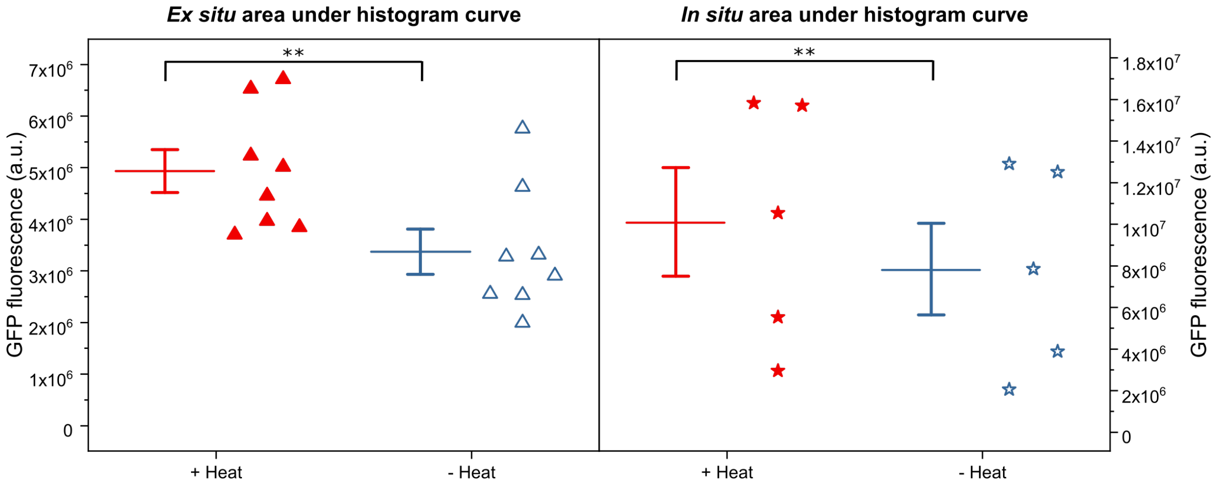

Figure S8. GFP expression as the area under the histogram curve obtained using flow cytometry. The total fluorescence of a sample can be calculated as the sum of each cell's fluorescence i.e. the area under the GFP expression histogram curve. Samples induced with artificial cells heated (red) *ex situ* (triangles, left) show a 1.5-fold increase in GFP fluorescence compared to samples induced using non-heated artificial cells (blue). The in-situ activation (stars, right) produced an area 1.3 times higher when the artificial cells were heated. Each data point corresponds to independent experiments. Solid lines represent the mean, error bars correspond to 1 S.E.M (n=8 or 5, *ex situ* and *in situ* respectively). P-value calculated using the 99.5 % confidence interval (**P < 0.005).

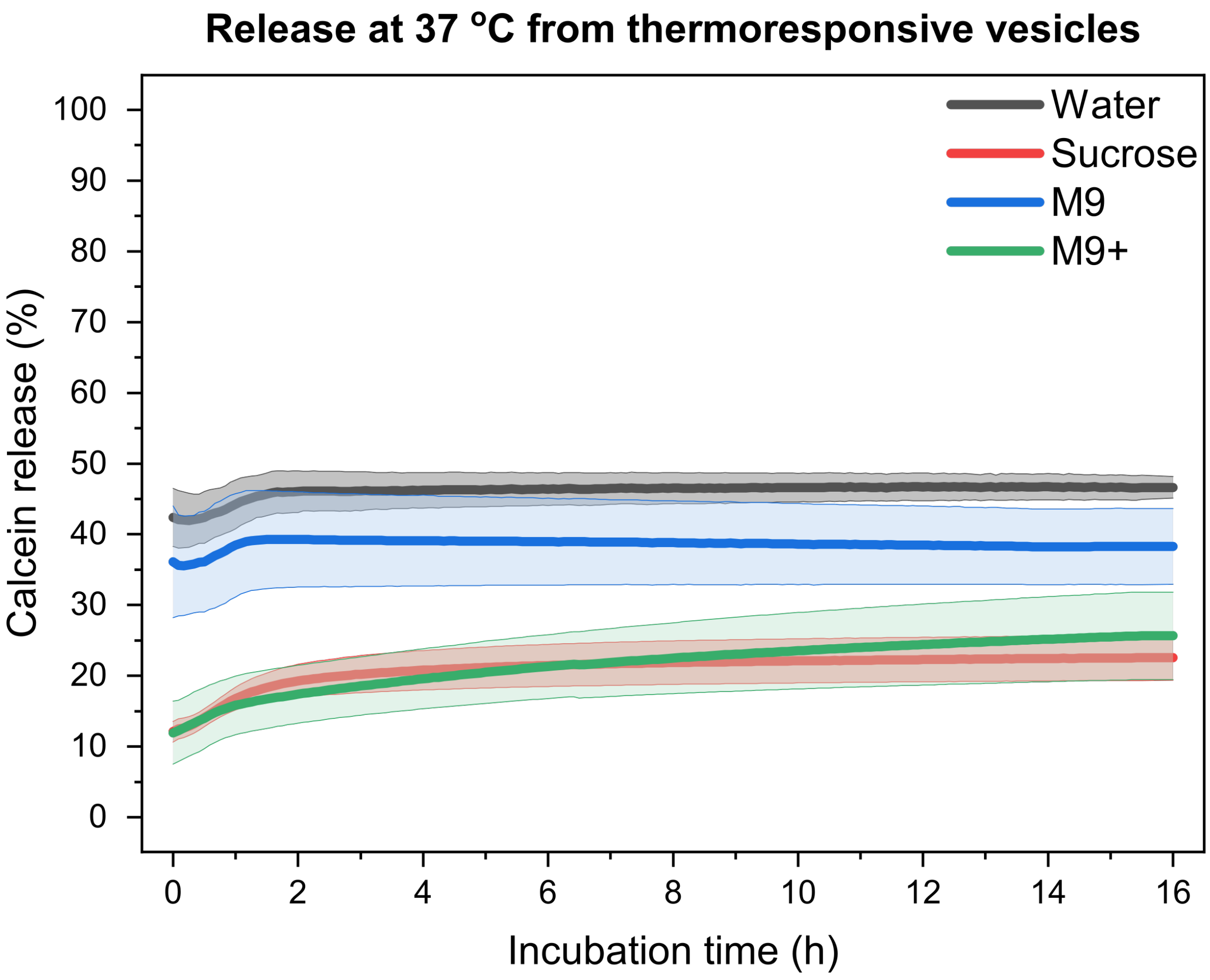

Figure S9. Passive release from calcein-loaded thermoresponsive artificial cells in different media at 37 °C. The osmolality of the media affects the passive release from artificial cells. Distilled water (black line) was employed as a control, its osmolality is 0 mOsm/kg and caused a 50 % passive calcein release. A 500 mM sucrose buffer (pH= 7.4, osmolality= 505 mOsm/Kg; red line) is generally employed as the control buffer, where no passive leakage occurs from calcein vesicles (internal calcein= 425 mOsm/kg). The media employed during vesicle-mediated protein expression experiments (M9; blue line) is hypoosmotic, 130 mOsm/kg, resulting in 40 % passive release being observed. To improve it, M9 was supplemented with more fructose to match the osmotic pressure of calcein (M9+, 423 mOsm/kg; green line). Calcein release in this M9+ buffer matches the release in sucrose – only a 10 % increase was detected over long incubation. New activation experiments were then performed using M9+ to reduce the passive leakage that causes expression in non-activated samples. The calcein release was calculated as the quotient of the fluorescent signal by the total fluorescence resulting from lysing the vesicles using triton. The release at time= 0 h for water and M9 is caused by a quick release when the vesicles are added to the media before starting to measure. The Shadowed area represents 1 SD (n=3).

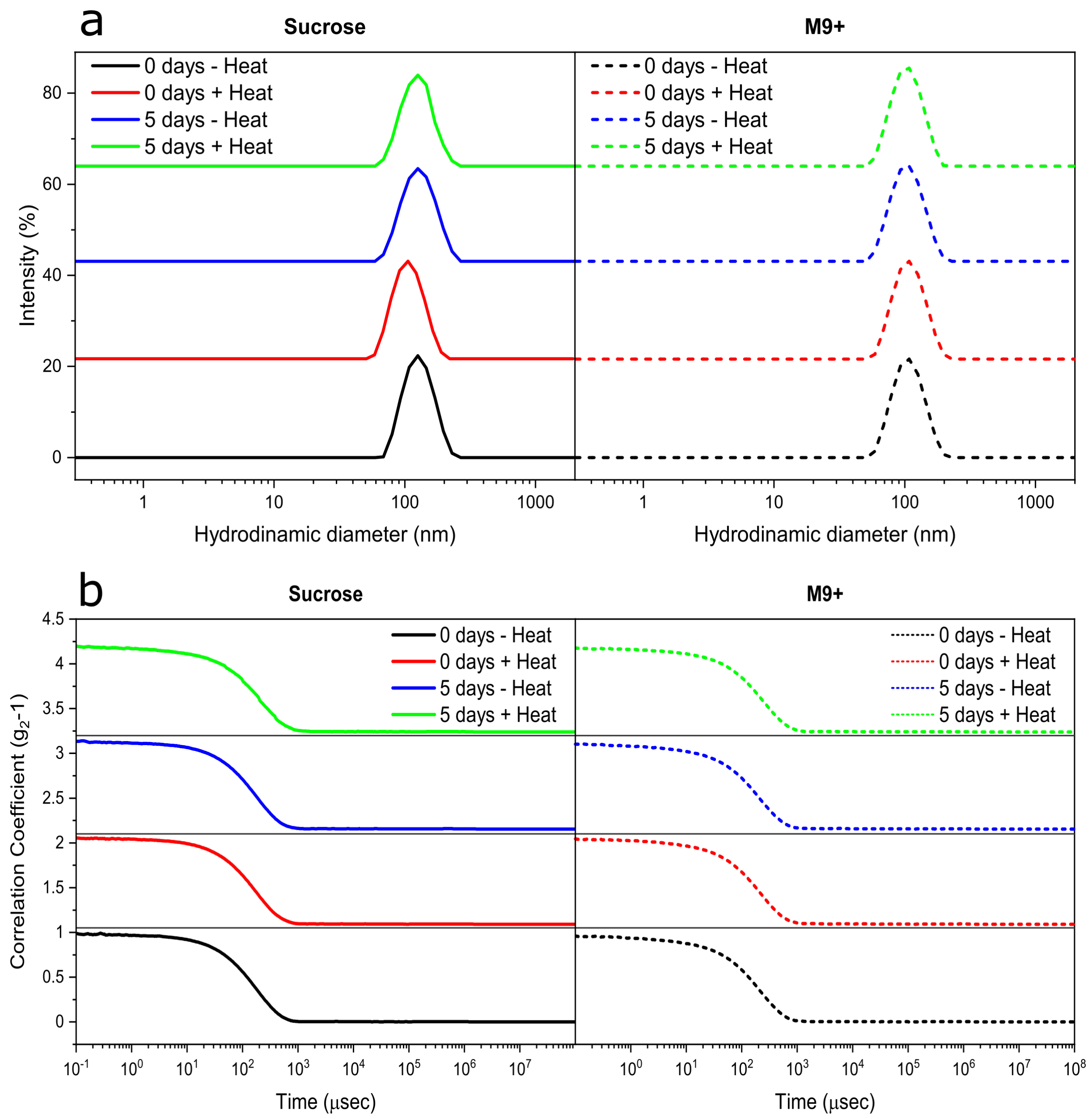

Figure S10. DLS measurements of artificial cells in the bacterial growth medium for temperature-controlled protein expression. **a)** Stability over 5 days is observed in samples diluted with 500 mM sucrose buffer (left) and in the growth medium (M9+, right). Both non-heated (black) and heated (red) vesicles maintained their mean diameters at 125.6 nm when in sucrose buffer and at 108 nm when in M9+ buffer. All the samples show a uniform size distribution as indicated by the polydispersity indexes: For non-heated samples, values started at 0.02 and 0.08 when in sucrose or M9+ just after preparation (day 0) and increased to 0.04 and 0.11 after 5 days (blue). For heated samples, the increase was from 0.06 to 0.12 in sucrose and from 0.07 to 0.09 in M9 (green)+. **b)** Correlograms of the DLS measurements show a single exponential decay that corresponds to mono-size particle dispersions.

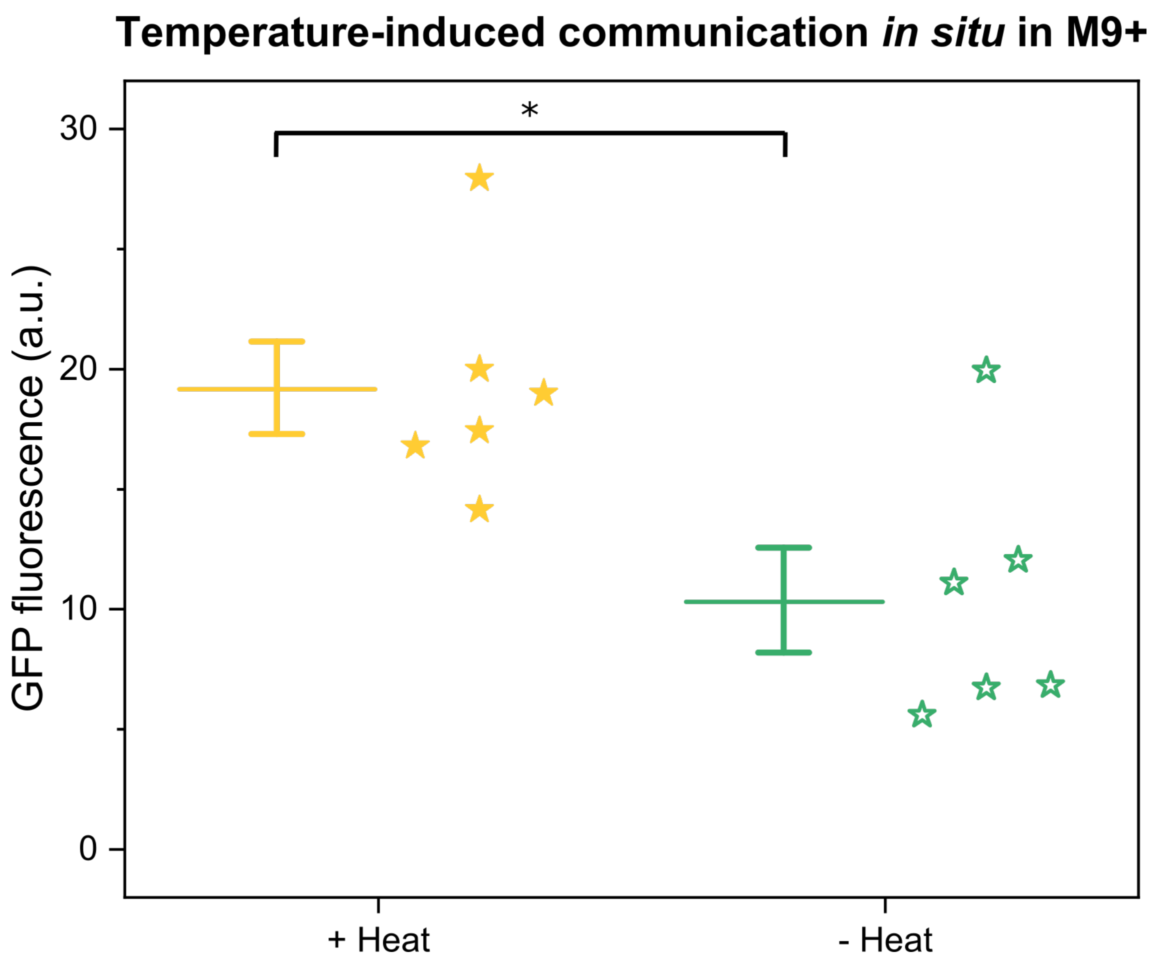

Figure S11. In-situ temperature artificial cell-mediated activation of GFP expression in M9+. The GFP expression with heated artificial cells (yellow stars) is 2 times higher than with non-activated artificial cells (green stars). Each data point corresponds to independent experiments. Solid lines represent the mean, error bars correspond to 1 S.E.M (n=6). P-value calculated using the 99.5 % confidence interval (*P<0.05).

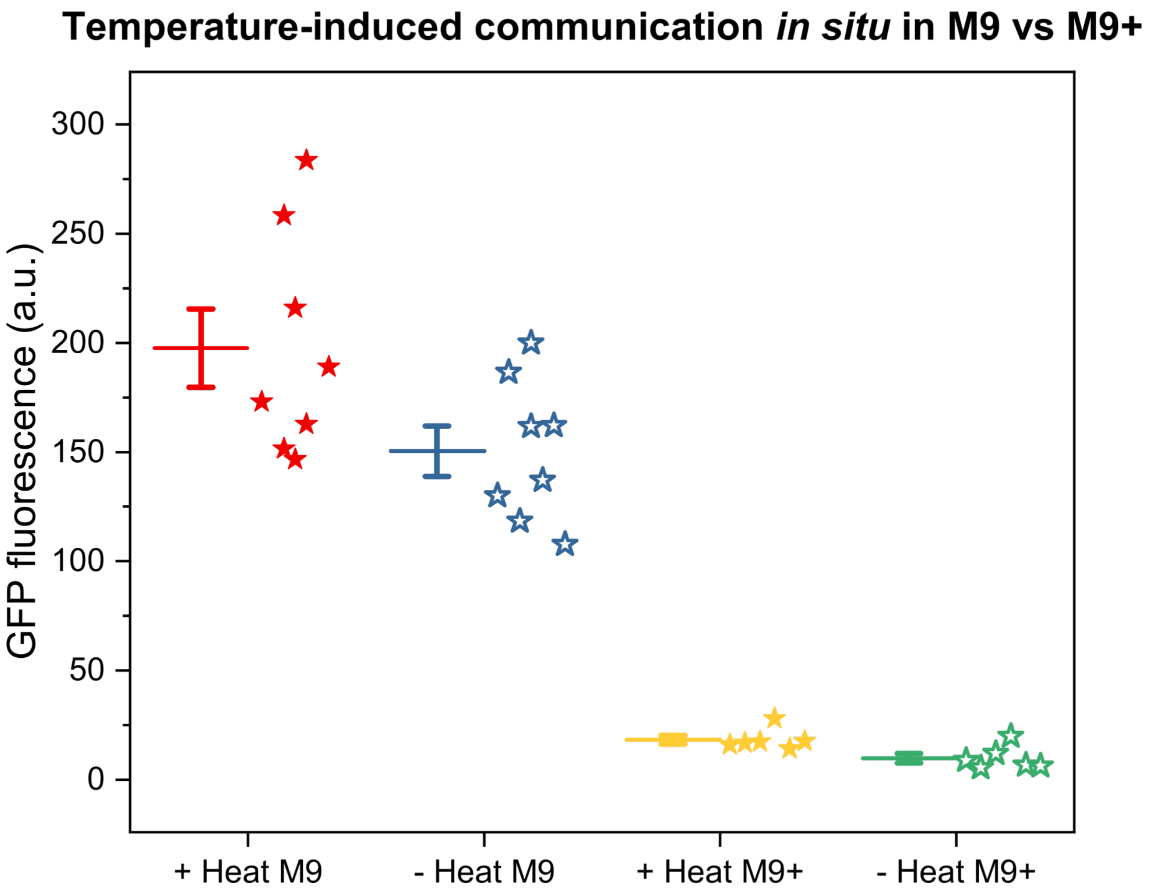

Figure S12. In-situ activation of temperature-controlled protein expression with artificial cells in M9 or M9+ as the expression medium. Despite the higher difference in GFP expression between activated (filled stars) and non-activated (empty stars) artificial cells in M9+ than in M9 (2-fold vs 1.3), the GFP fluorescence is much lower when using M9+. Each data point corresponds to independent experiments. Solid lines represent the mean, error bars correspond to 1 S.E.M (n=8 for M9 samples, n=6 for M9+ samples).

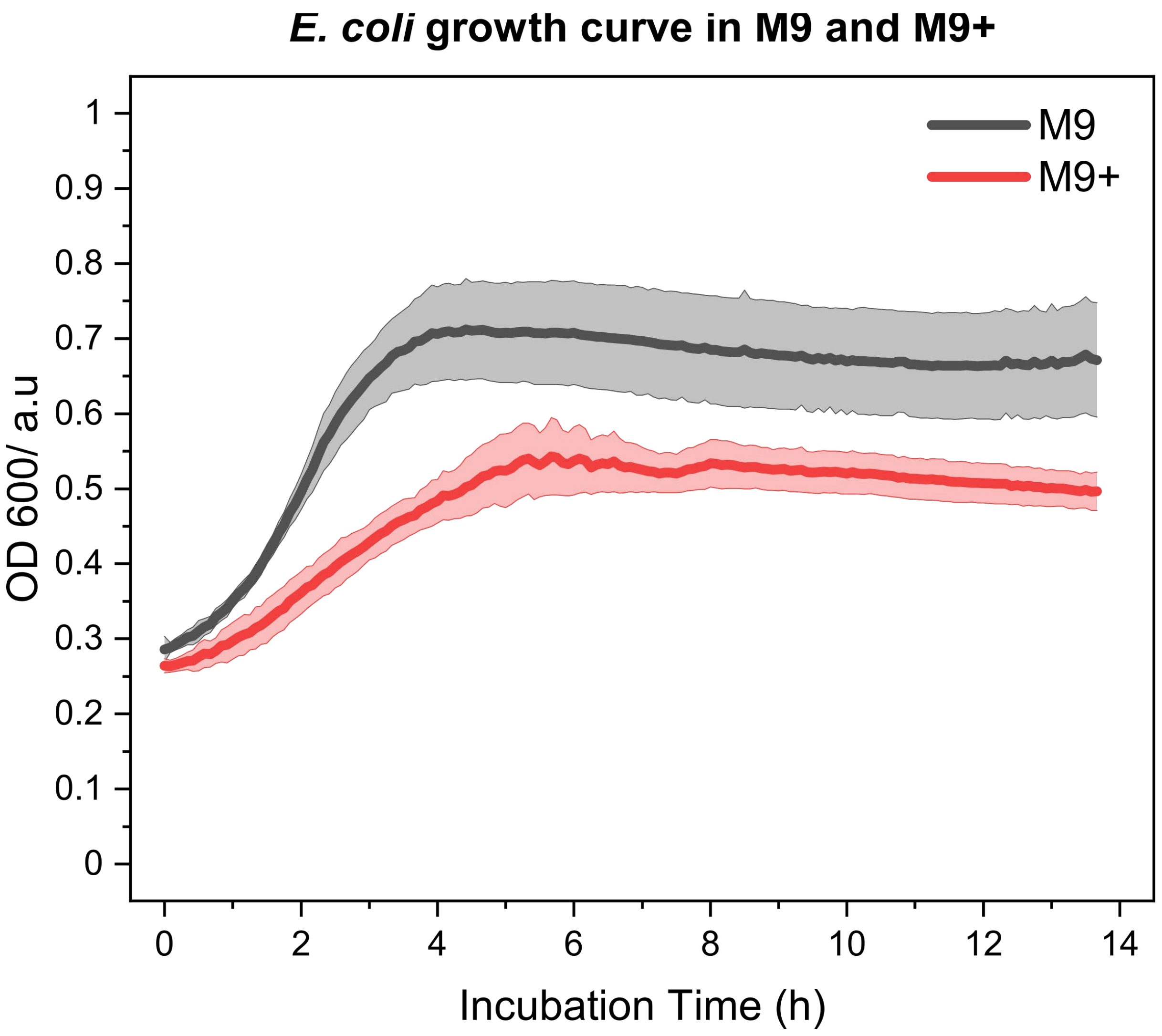

Figure S13. *E. coli* growth curve at 37 °C in M9 (black) and M9+ (red). The addition of fructose to M9, to yield an isosmotic media to the rhamnose encapsulated within artificial cells, results in a decrease in bacterial growth. M9+ is isosmotic to the encapsulated rhamnose buffer but hyperosmotic to the bacterial cells. The Shadowed area represents 1 SD (n=3).

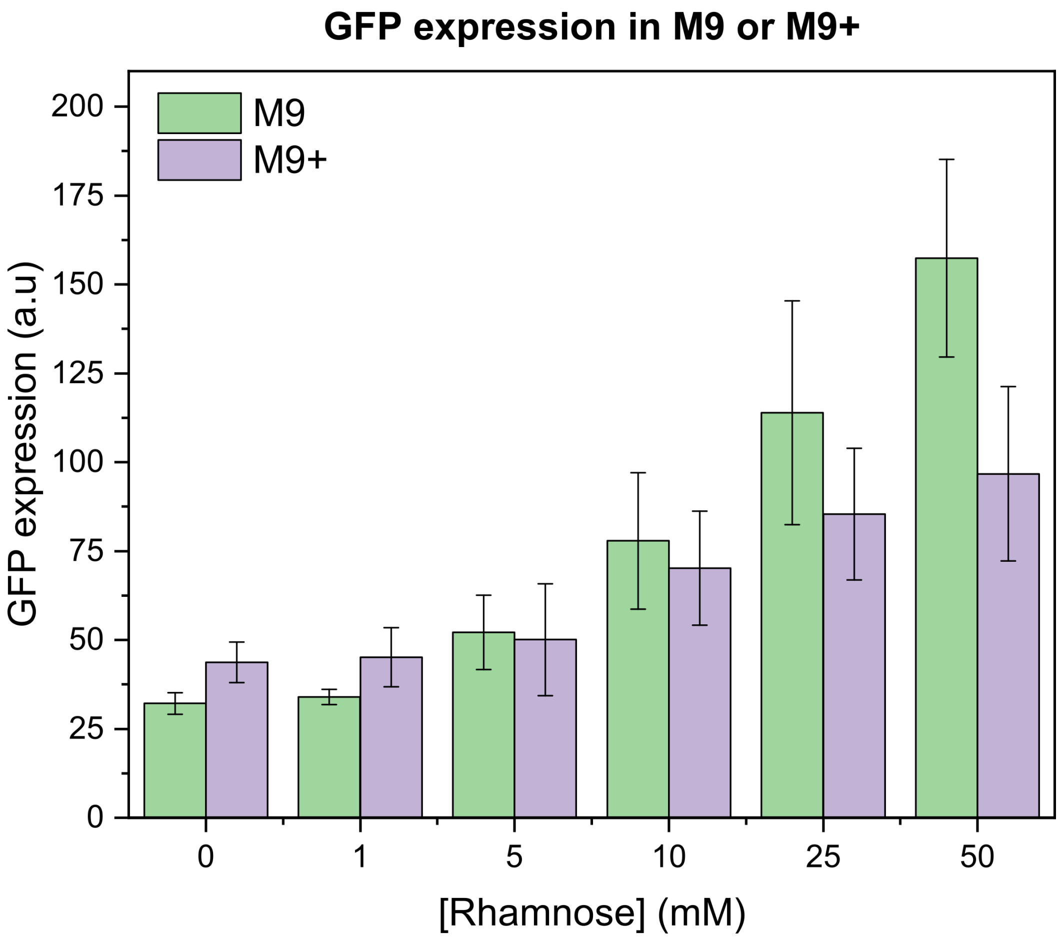

Figure S14. The protein expression in *E. coli* is affected by the osmolality of the growth medium. After 4 h of incubation at 37 °C, the GFP expression using standard concentrations of rhamnose is lower when using M9+ (purple) than with M9 (green). Error bars show 1 SD. (n = 3).

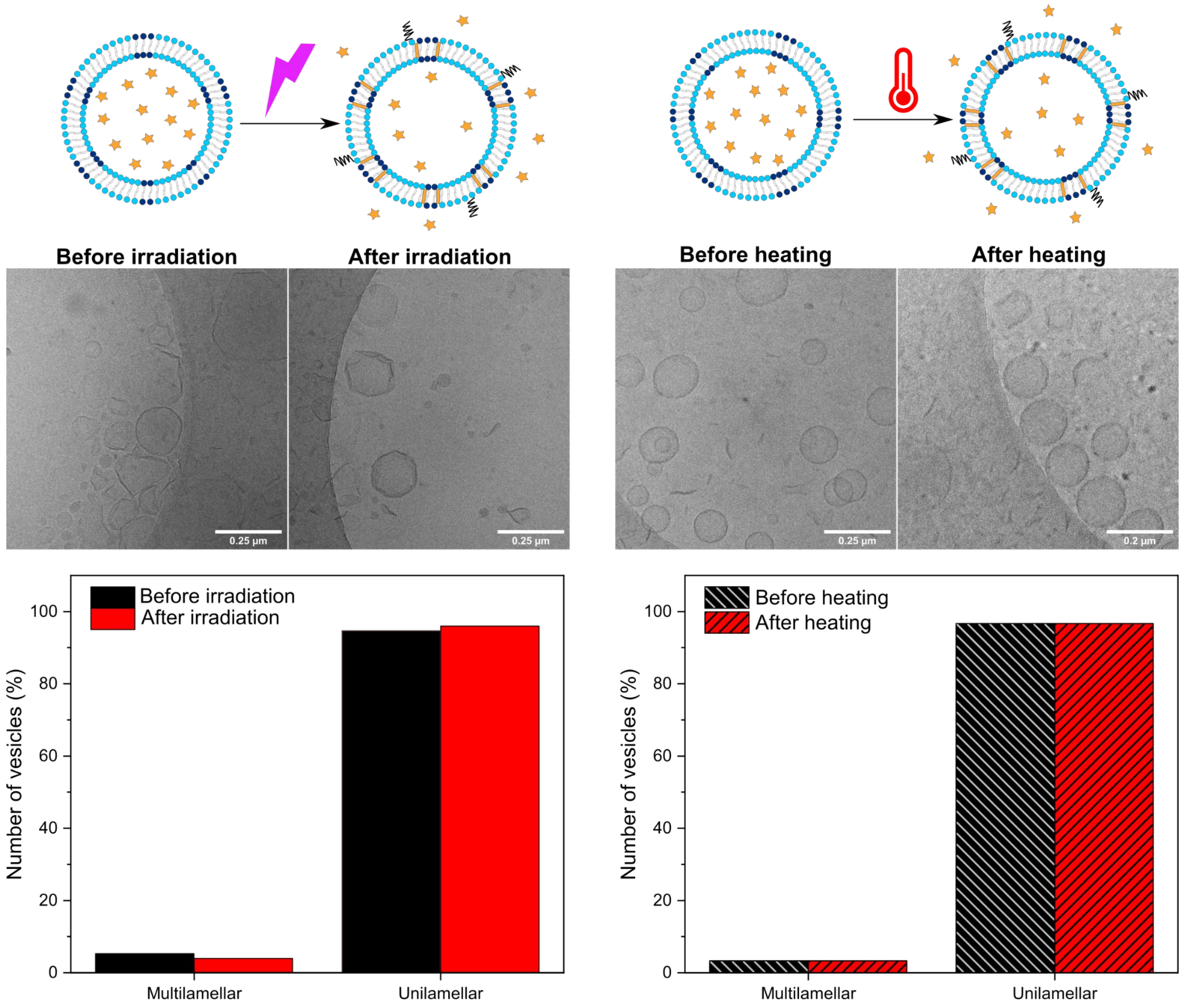

Figure S15. Cryo-TEM analysis of stimuli-responsive artificial cells. All the samples were predominantly unilamellar (~ 95 %) as determined by analysing light-responsive (left) or thermoresponsive (right, dashed columns) individual vesicles before (black) or after (red) the stimulation (40 min irradiation with 254 nm light, or heating at 43 °C during 5 min). The light-responsive composition yielded faceted vesicles independently of the irradiation due to the presence of segregated $DC_{8,9}PC$ domains. Whereas the thermoresponsive vesicles were mostly spherical. (n=150 vesicles for each sample).

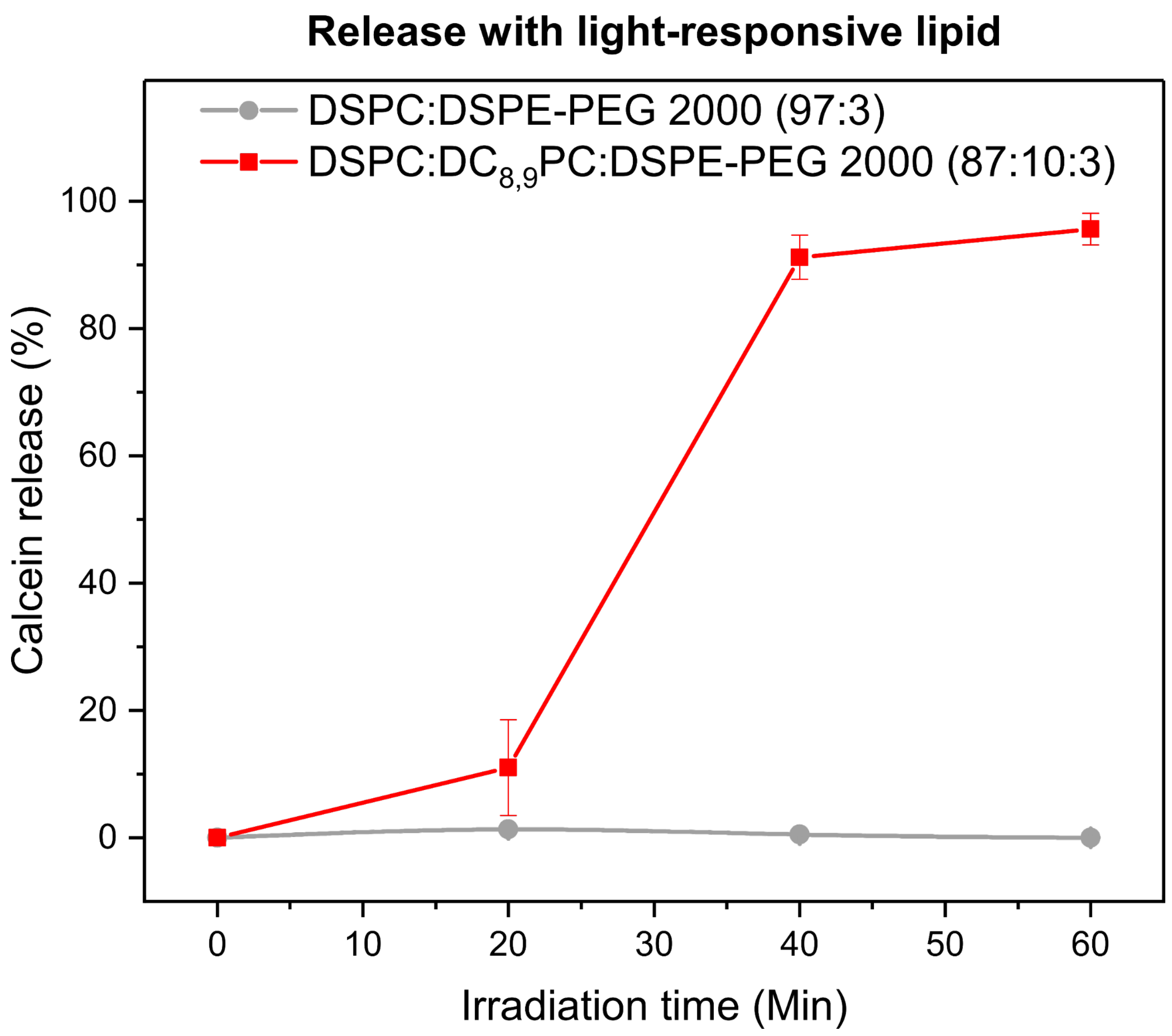

Figure S16. Release produced by the light-responsive lipid $DC_{8,9}PC$. $DC_{8,9}PC$ is required to induce content release with UV irradiation. Comparison of release assays with $DC_{8,9}PC$ (red) and without $DC_{8,9}PC$ (grey). Composition without $DC_{8,9}PC$ was DSPC:DSPE-PEG 2000 (97:3) and showed no leakage (max 1.3 %). Error bars 1 S.D (n=3).

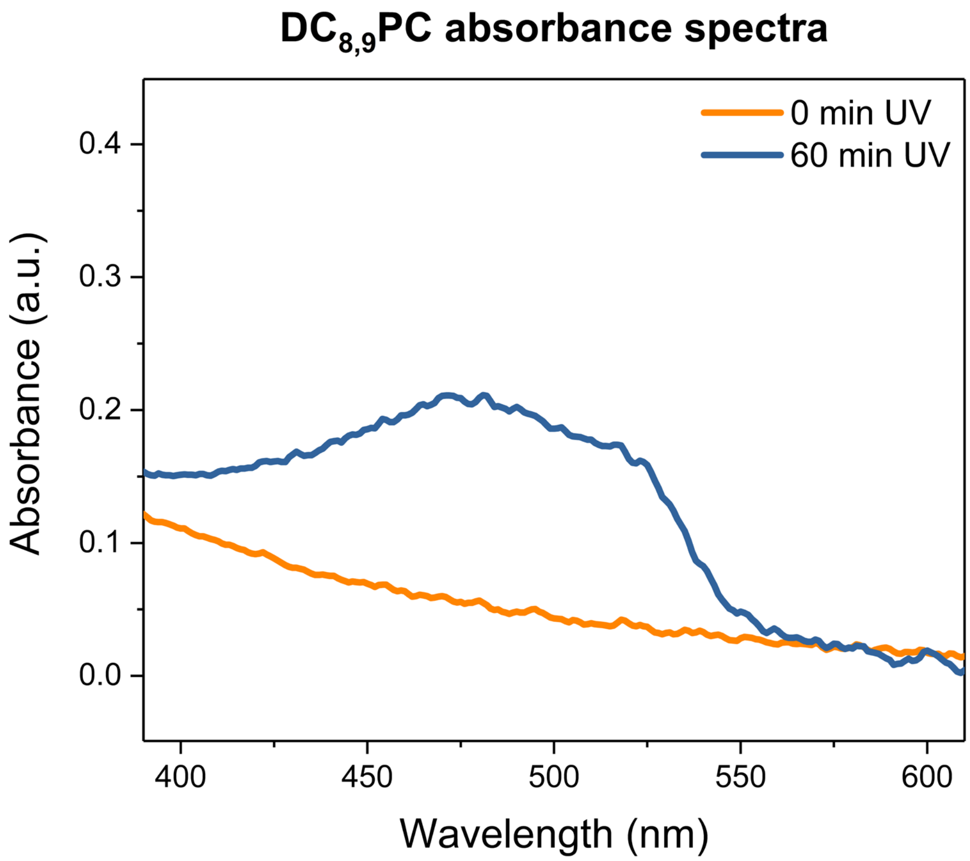

Figure S17. Absorbance spectra of the artificial cells before and after irradiation. Photopolymerisation results in the formation of ene-yne conjugates which causes a broad peak to appear in the 440-540 nm region of the absorbance spectrum. Its maximum at 475 nm was used to assess the extent of polymerisation with different irradiation times for Fig. 4 a.

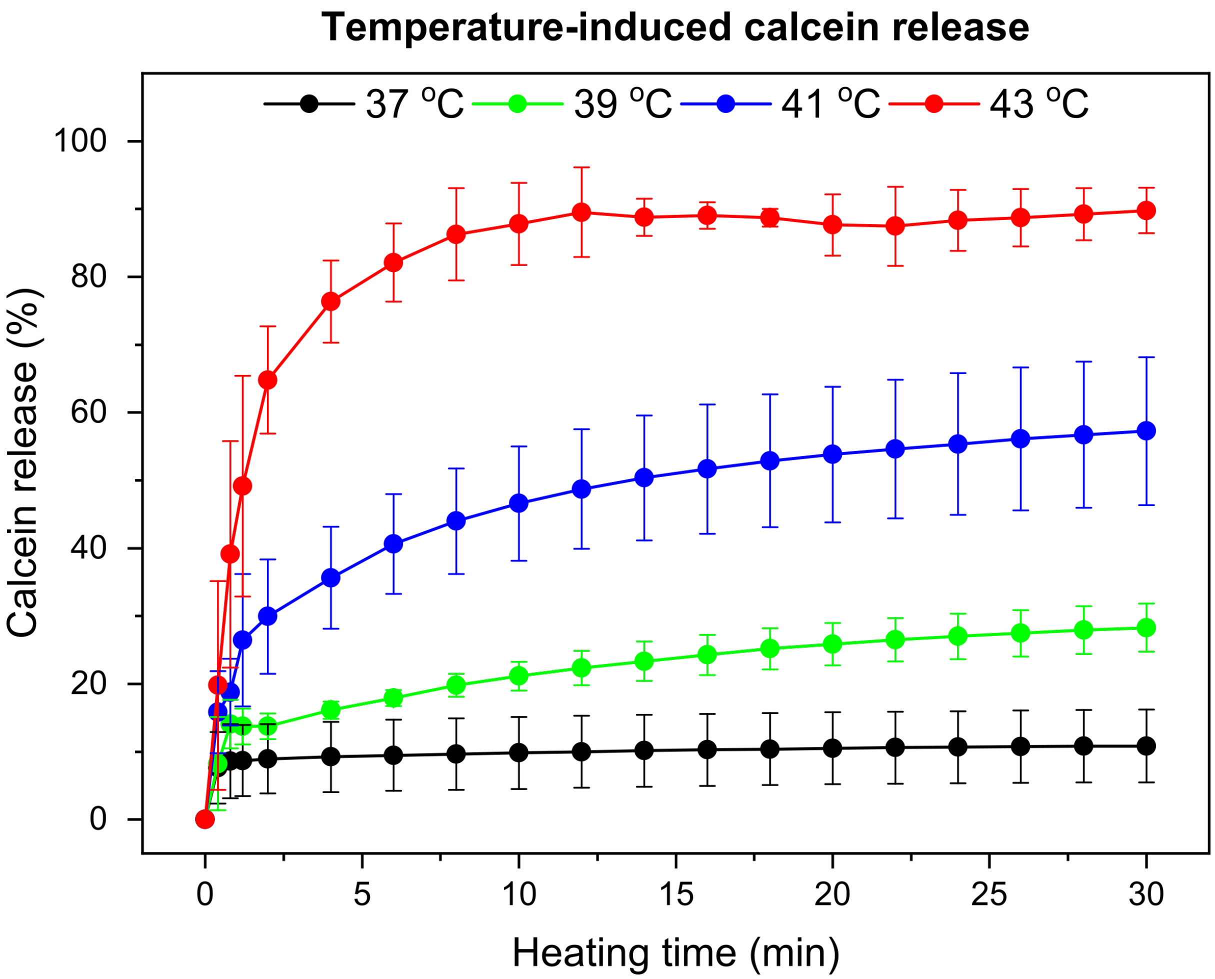

Figure S18. Calcein release from thermoresponsive vesicles at different temperatures. While at 37 °C (black) only 8 % of calcein releases, higher temperatures enable a higher release. This further release occurs faster and in a greater amount. Even when heated for longer, none of the temperatures (37, 39 or 41 °C; black, green or blue line) tested reached the same level of release as 43 °C (red). Error bars 1 S.D (n=3).

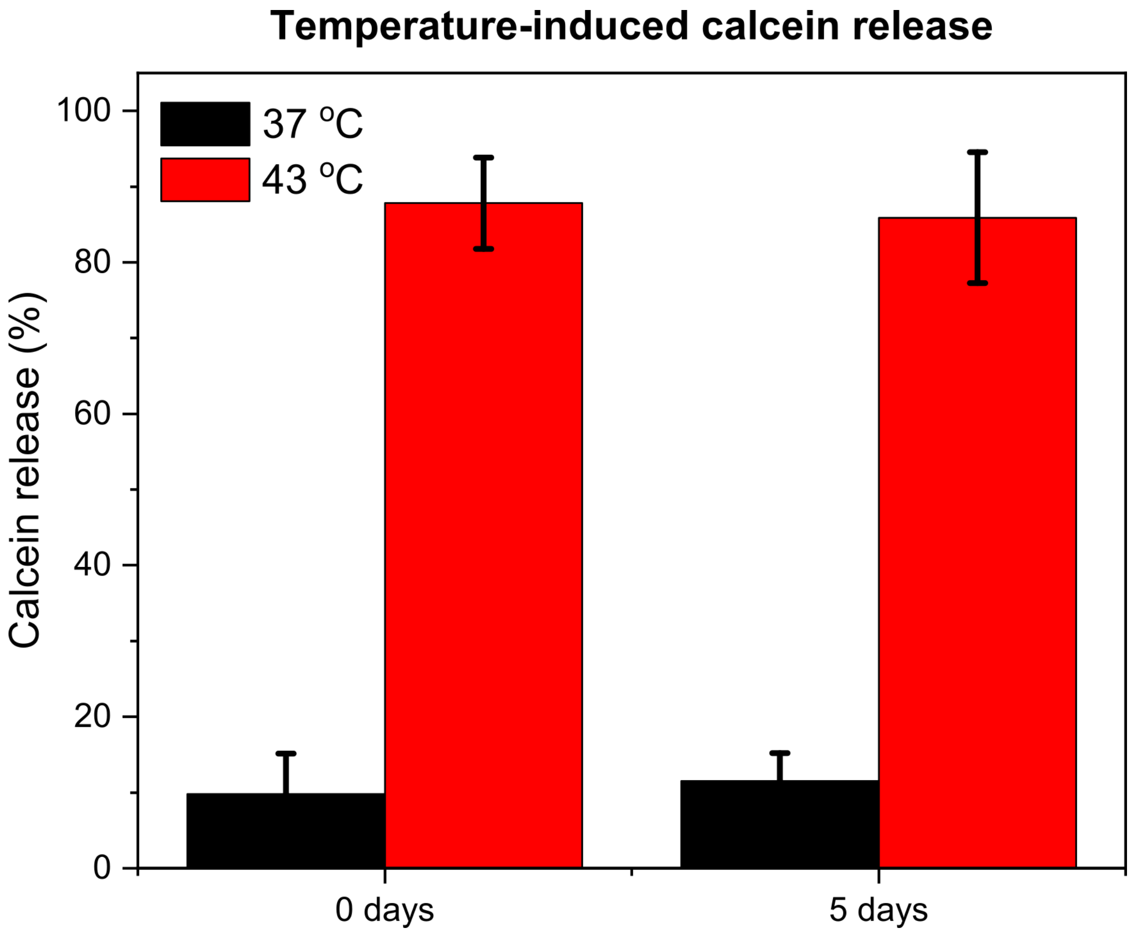

Figure S19. Temperature-induced calcein release from artificial cells after 5 days of storage. Calcein release was measured after 10 min of heating at 43 °C (red) or 37 °C. Then the artificial cells were stored for 5 days at 4 °C, and the release assay was repeated. No difference was observed in the release efficiency. Error bars show 1 SD, (n = 3).

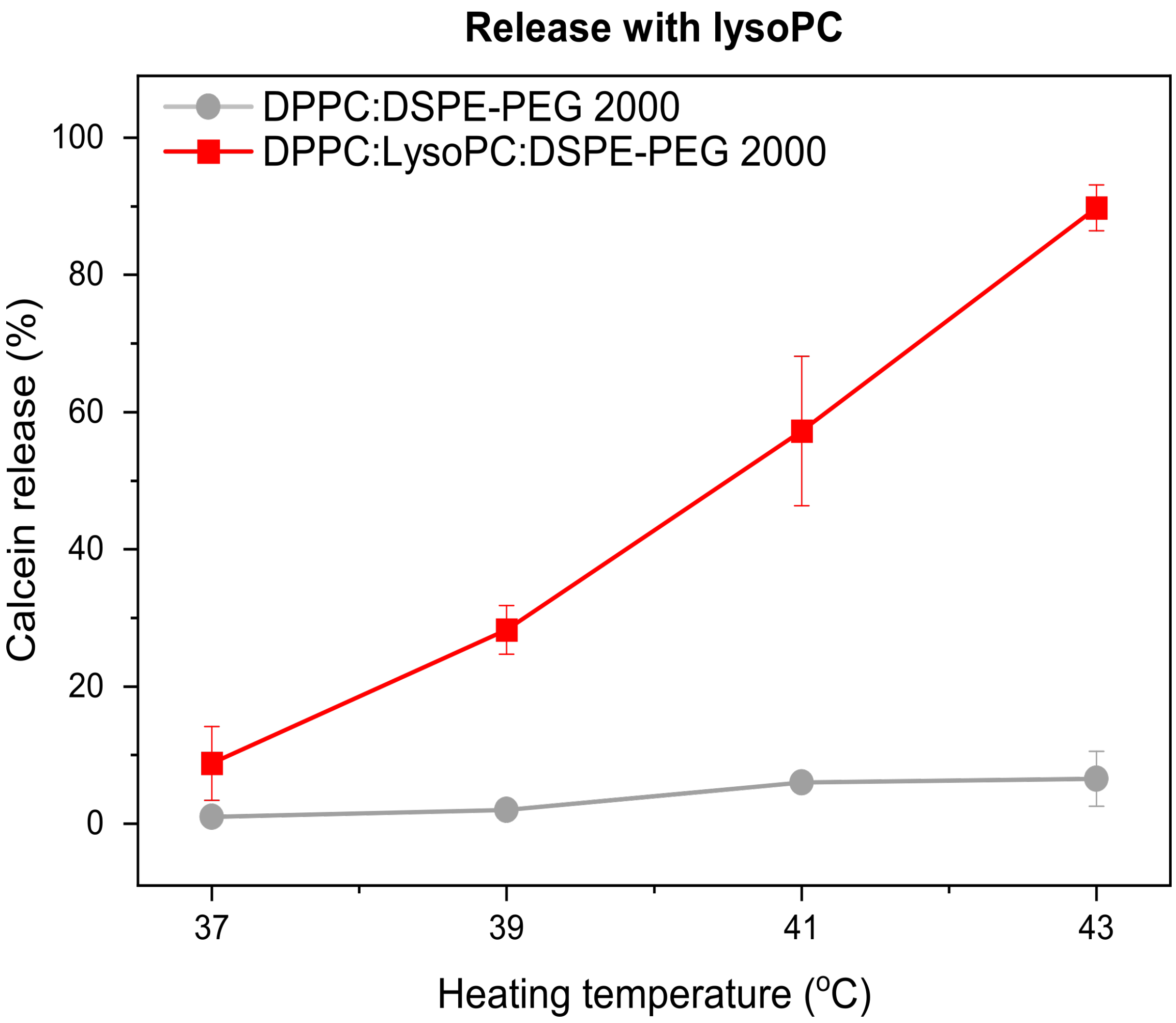

Figure S20. Release produced by incorporating lysoPC. LysoPC greatly enhances the temperature-mediated calcein release. Comparison of release after 30 min of heating at different temperatures with lysoPC (red) and without lysoPC (grey). The composition without lysoPC -DPPC:DSPE-PEG 2000 (96.2:3.8)- showed a maximum leakage of 8 %. Error bars 1 S.D (n=3).

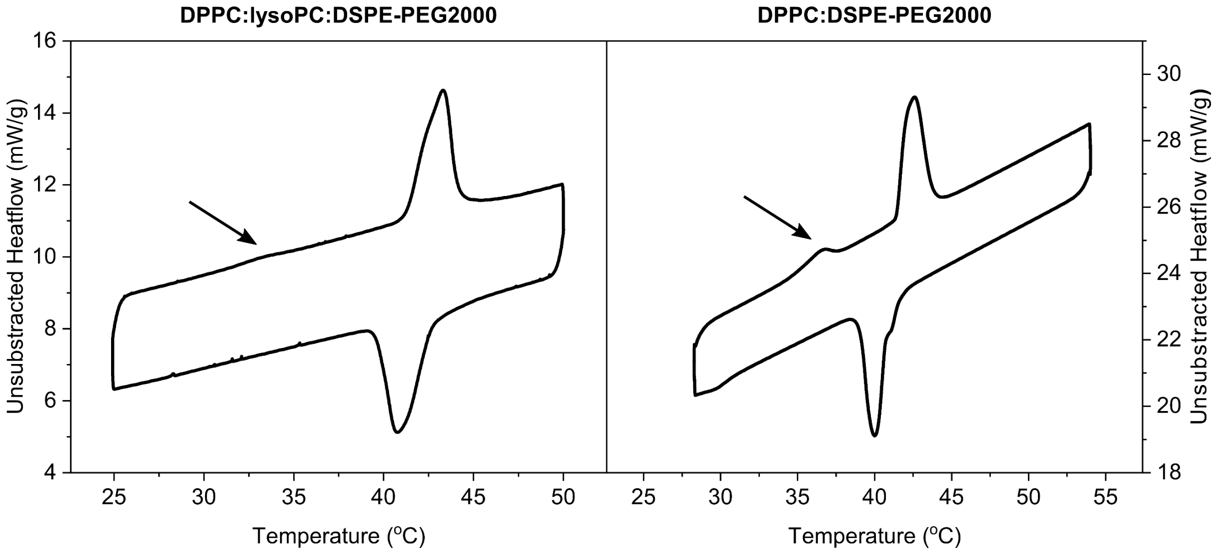

Figure S21. Thermogram of DPPC:lysoPC:DSPE-PEG 2000 and DPPC:DSPE-PEG 2000. The incorporation of 9.6 % of lysoPC results in the disappearance of the pre-transition at ~37 °C (indicated with arrows). The main transition of DPPC at 41 °C is not affected by the incorporation of lysoPC, however, the release is drastically enhanced due to the stabilisation of transient pores at the grain boundaries.

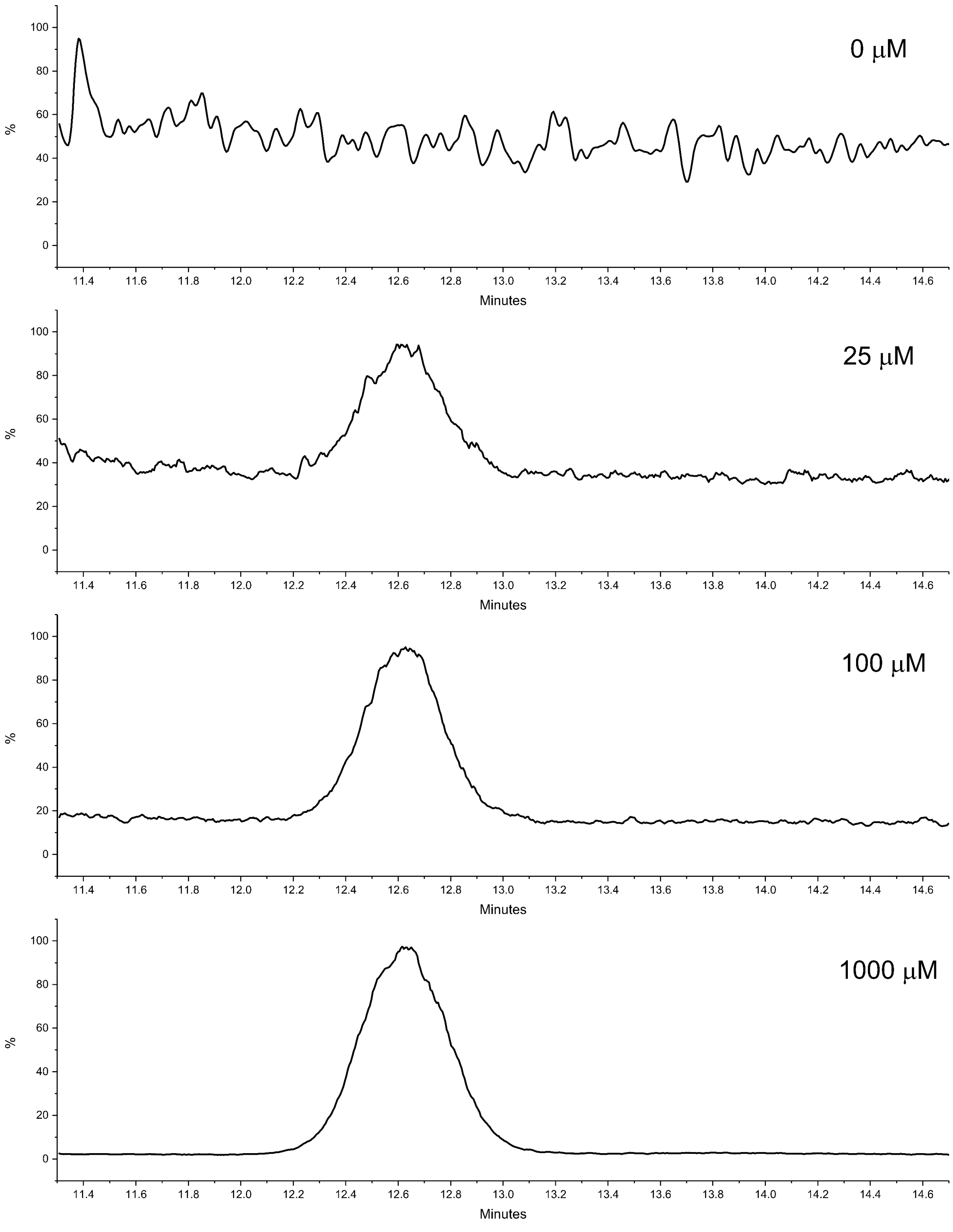

Figure S22. Chromatograms obtained using standard concentrations of IPTG. IPTG eluted at 12.60 min and was detected as $[M+H]^+$=239. The areas under the curve were used to create a calibration curve.

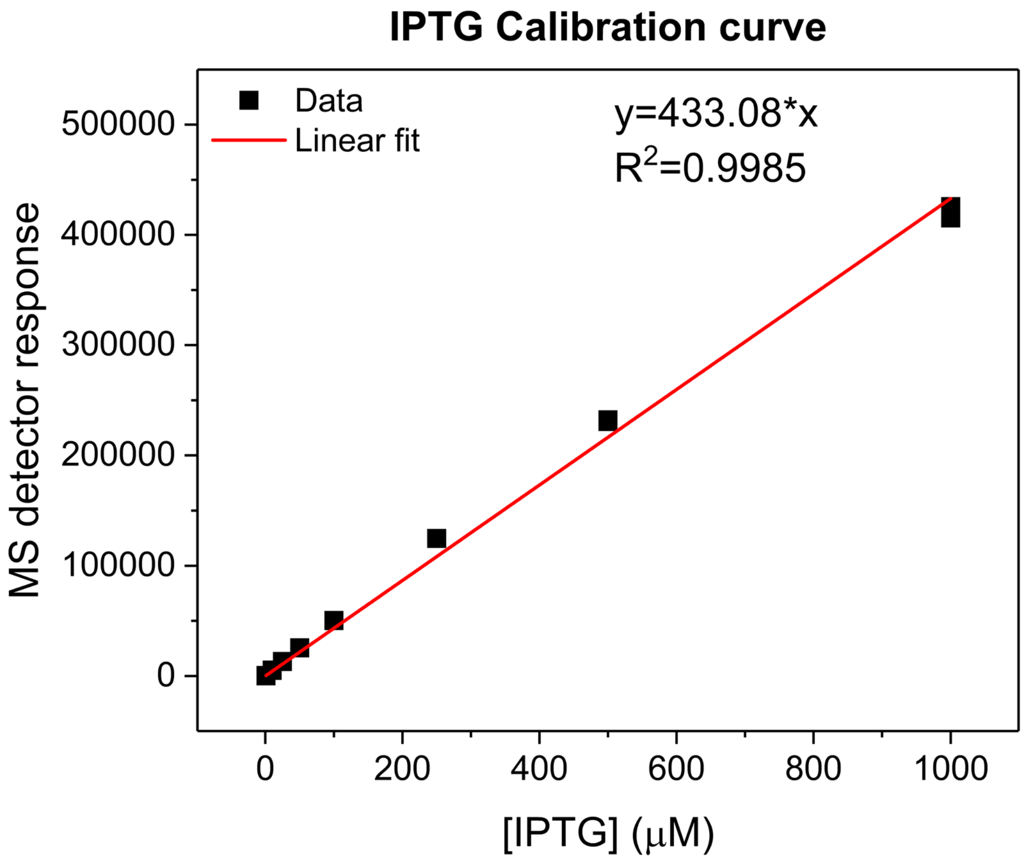

Figure S23. IPTG calibration curve prepared using LC-MS. Constructed employing the area under the curve of the chromatograms obtained using standard concentrations of IPTG from 1 to 1000 µM measured at $[M+H]^+$=239.

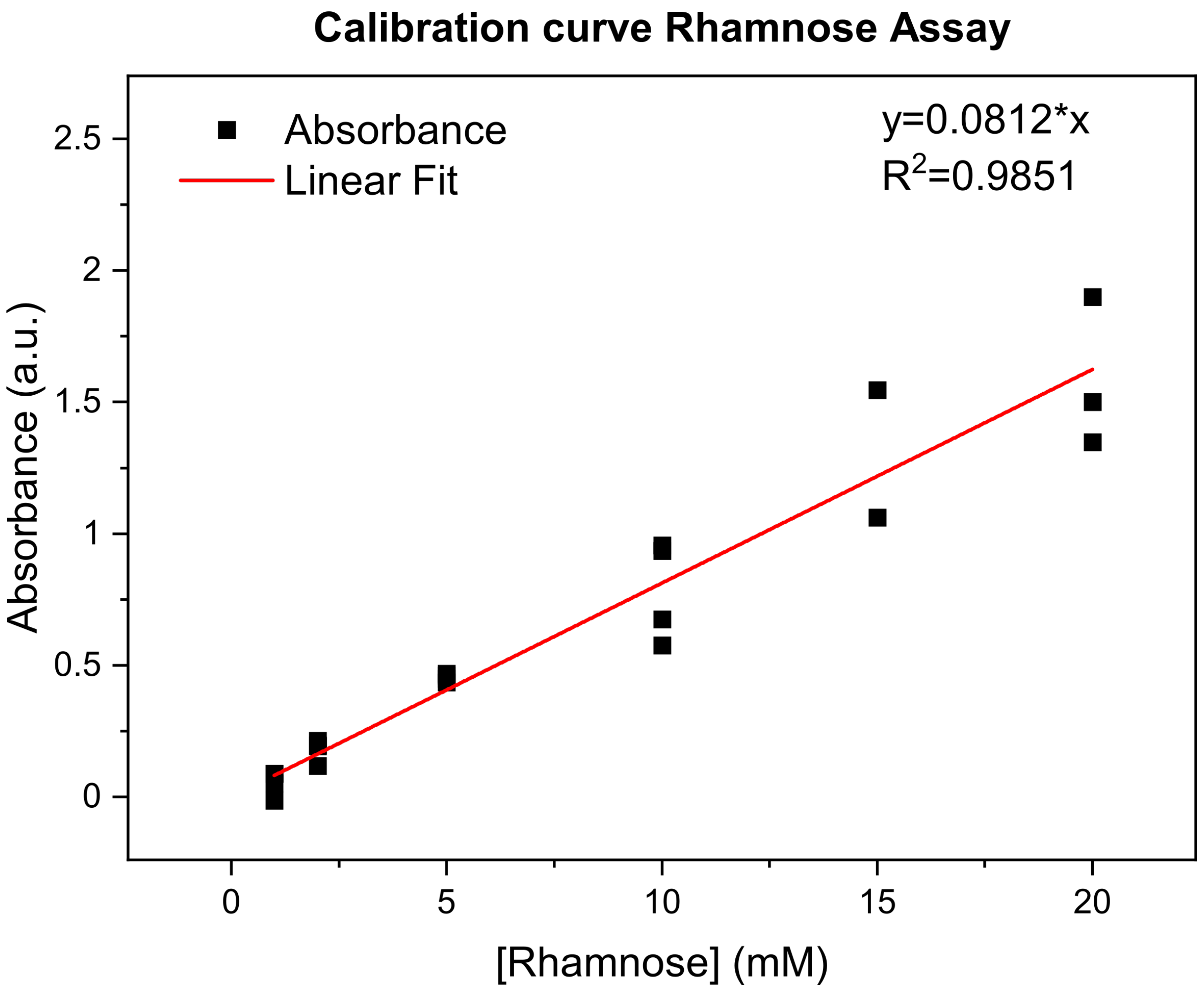

Figure S24. Rhamnose calibration curve prepared using a rhamnose enzymatic kit. NAD^+ oxidises rhamnose, yielding NADH, which is measured at 340 nm by UV-Vis spectroscopy after 10 min of reaction. Standard concentrations of rhamnose from 1 to 20 mM were employed to create this calibration curve which was later employed to determine the unencapsulated rhamnose after SEC purification of thermoresponsive artificial cells.

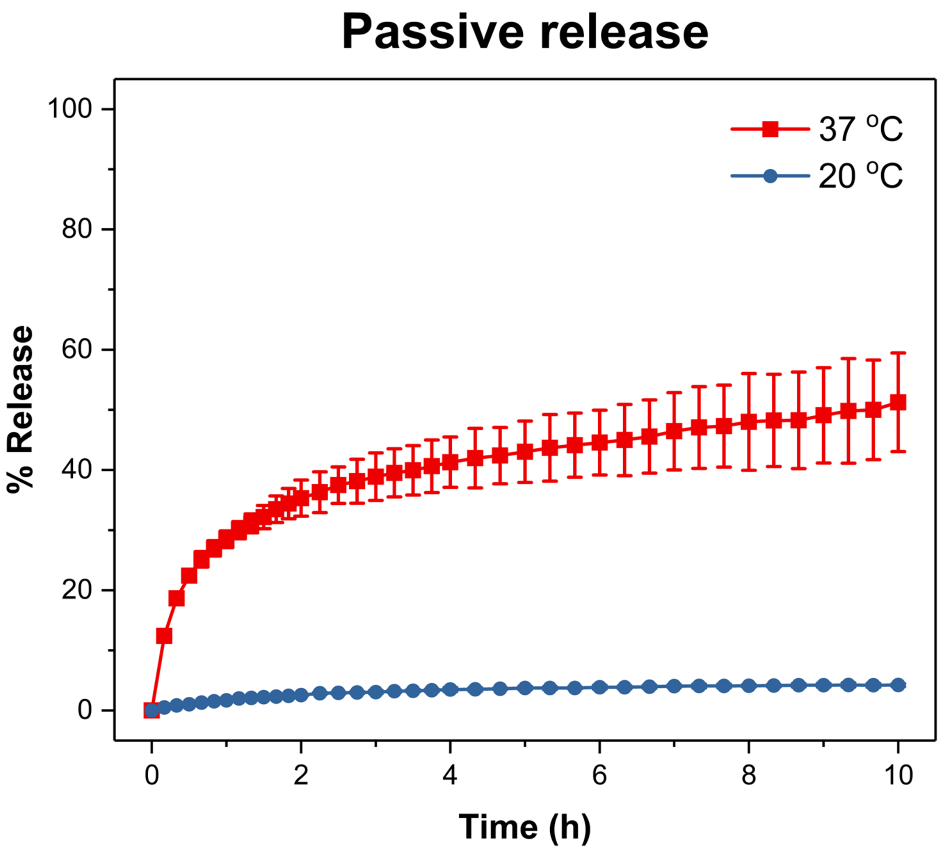

Figure S25. Temperature-induced release using the light-responsive composition. Passive release from calcein-loaded artificial cells. At 37 °C (red) after 1 h, there was a ~30 % release that raise to ~50 % after 10 h. Meanwhile, the fluorescence at 20 °C (blue) only increased a 5 % in 10 h. Protein expression was performed at 20 °C to ensure the activation is produced by light irradiation.

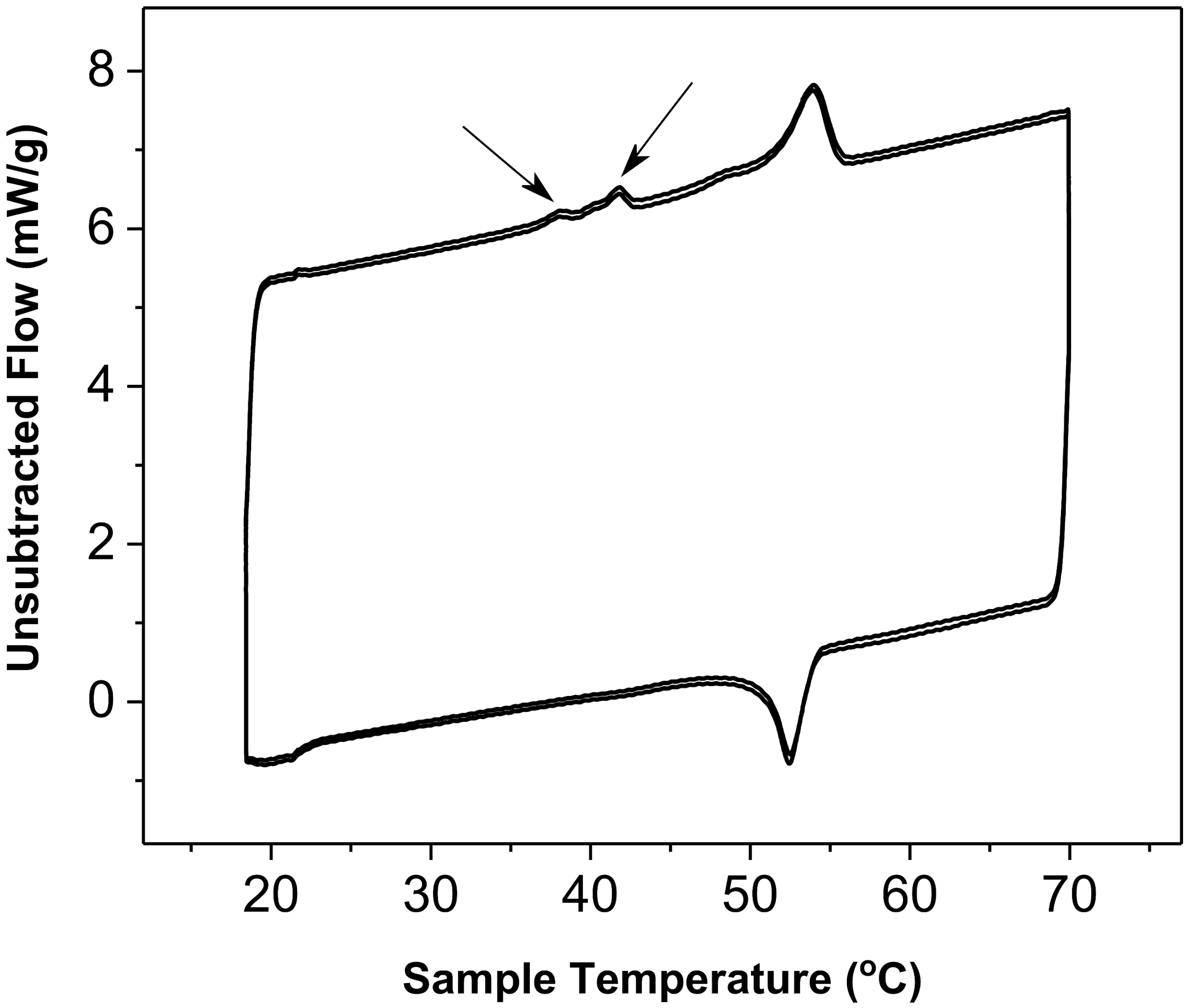

Figure S26. Thermogram of DSPC:$DC_{8,9}$PC:DSPE-PEG 2000. Apart from the main transition at ~53 °C, corresponding to DSPC, two pre-transitions associated with $DC_{8,9}$PC were found at 37 °C and 39 °C (indicated with arrows).
